# Chaotic and dynamic vibration analysis of a time-delayed nonlinear mathieu oscillator via non-perturbative approach

**DOI:** 10.1038/s41598-026-45062-7

**Published:** 2026-04-13

**Authors:** Galal M. Moatimid, T. S. Amer, Yasmeen M. Mohamed

**Affiliations:** 1 Department of Mathematics, Faculty of Education, Ain Shams University, Roxy, Cairo, Egypt; 2 Department of Mathematics, Faculty of Science, Tanta University, Tanta, 31527 Egypt

**Keywords:** Nonlinear vibration, Dynamic stability, Time delay, Non-perturbative approach, He’s frequency formulation, Bifurcation and chaos, Analytical–numerical validation, Engineering, Mathematics and computing, Physics

## Abstract

The study compares van der Pol and Rayleigh oscillators with time-delayed effects, providing insights into stability, bifurcations, and organization in nonlinear systems. It emphasizes unique dynamical properties and resonance conditions, strengthening the theoretical basis of the design of delay-controlled oscillatory systems. The aim is to adopt the non-perturbative approach, which transforms a weakly nonlinear oscillator of an ordinary differential equation into a linear one. Computed through a refined series approximation, the response manages both small and large oscillatory amplitudes without constraining assumptions, avoiding reliance on small parameter expansions. Validation outcomes reveal a strong agreement between parametric clarifications and the original nonlinear model, confirming the credibility of the proposed framework. Furthermore, a comprehensive assessment of the system’s stability is conducted in diverse situations. Two different instances of nonlinear Mathieu oscillators are inspected. A comprehensive stability analysis is performed for two nonlinear Mathieu-type oscillators with van der Pol and Rayleigh damping. Numerical simulations are carried out employing time histories, phase portraits, Poincaré maps, bifurcation diagrams, and Lyapunov exponents. These simulations ensure the accuracy and reliability of the analytical predictions. The outcomes expound distinct and contrasting stability performances for the van der Pol and Rayleigh oscillators. They demonstrate that nonlinear damping, excitation amplitude, natural frequency, and time delay play vital roles in governing the system’s dynamic response. It is observed that stability generally decreases with increasing natural and excitation frequencies, while it enhances with higher damping and excitation amplitudes. Moreover, the van der Pol and Rayleigh oscillators exhibit opposite impacts of nonlinear damping and natural frequency on stability. The novelty of this study lies in employing a non-perturbative approach to time-delayed Mathieu-type oscillators. The obtained findings provide valuable physical insight and practical guidance for the analysis and design of delay-controlled oscillatory systems in mechanical, structural, and engineering implementations.

## Introduction

The Mathieu oscillator originates from Mathieu’s equation and plays a significant role in classifying stability and instability zones in parametrically excited systems, with wide-ranging implementations in physics, engineering, and electronics. The stability of such systems is commonly characterized through Floquet or characteristic exponents associated with the fundamental solution matrix^1^. As a consequence, the determination of stability constraints of Mathieu oscillators has long been a central topic in the analysis of nonlinear dynamical systems. Diverse analytical approaches are developed to get periodic and approximate solutions of Hill-type and Mathieu oscillators. Early investigations proposed analytical periodic approximations to characterize parametric resonance and instability areas^2^. These contributions established a foundation in understanding the dynamic response of parametrically excited systems under idealized propositions. The inclusion of time-delay influences introduces additional complexity into nonlinear oscillatory systems. Often causing delay-induced resonance, amplitude amplification, and modulated stability characteristics. Former investigations have shown that time-delay terms can significantly alter oscillatory performance and induce long-lasting oscillations under external excitation^3^. The combined impact of damping, forcing, and delay has been explored in diverse nonlinear oscillators, including time-delayed Duffing-type systems^4^.

Nonlinear oscillators incorporating additional influences such as fractional damping, stochastic excitation, coupling, and time-delayed feedback were widely explored to better represent realistic physical systems^5–8^. These investigations demonstrate that nonlinear damping mechanisms and delay feedback significantly influence resonance performance, bifurcation structures, and stability characteristics across diverse dynamical models. Most analytical studies of Mathieu-type and time-delayed oscillators rely on perturbation techniques such as the multiple time scales method (MTSM), Lindstedt–Poincaré approaches, and related asymptotic methods, typically developed under assumptions of weak nonlinearity and near-resonant conditions^9–16^. Alternative frameworks, including the NPA method, have also been introduced to analyze parametrically damped nonlinear Mathieu–Duffing systems and their stability characteristics. Beyond their theoretical significance, Mathieu-type oscillators exhibit substantial practical importance, as outlined below:

- Mathieu oscillators are widely employed to assess the stability of mechanical systems subjected to periodic excitations, such as vibrations in structural beams, suspension bridges, and rotating machinery.
- Their ability to distinguish stable and unstable operating zones is essential in predicting and preventing resonance-induced failures in engineering structures.
- The nonlinear dynamics of Mathieu-type systems support the development of advanced control strategies in robotics and mechatronics, particularly of systems affected by periodic excitations or time-varying factors.
- Mathieu-type models play a fundamental role in describing energy bands and band gaps in semiconductor and quantum devices governed by periodic potentials.
- In biological and biomedical implementations, Mathieu-based formulations are employed to model systems under periodic stimuli, aiding in the nonlinear classification of normal and pathological states.
- In aerospace engineering, Mathieu-type oscillators assist in predicting and suppressing oscillatory instabilities in aircraft wings and satellite structures, thereby improving structural integrity and flight safety.

The MTSM, as a cornerstone of classical perturbation theory, fundamentally relied on the assumption of a small parameter^1^. While perturbation techniques are widely employed to analyze nonlinear oscillators, obtaining exact analytical solutions remains challenging due to the inherent complexity of nonlinear ODEs. Alternative semi-analytical approaches, such as the homotopy perturbation method (HPM), were proposed to derive asymptotic solutions under weak excitation conditions; however, their dependence on small parameters can cause diminished accuracy outside limited regimes^17^. These limitations motivate the exploration of analytical frameworks that remain valid without restrictive small-parameter assumptions. Various semi-analytical techniques have been proposed to approximate the dynamic response of nonlinear oscillators. Among them, He’s frequency formulation (HFF), introduced by Ji-Huan He, was employed to estimate amplitude–frequency relationships with relatively low computational contribution^18–21^. Although HFF has demonstrated the effectiveness of certain classes of nonlinear oscillators, its accuracy depends on the choice of appropriate trial functions and evaluation points, which may limit its applicability in complex delayed and strongly nonlinear systems^22–25^.

Various analytical and semi-analytical methods are proposed to study nonlinear oscillatory systems, involving the HFF, which is employed to estimate amplitude–frequency relationships with relatively low computational contribution^26^. Nevertheless, many of these approaches rely on restrictive presumptions that limit their applicability to complex or strongly nonlinear systems. In recent years, the NPA has attracted escalating attention as a powerful alternative in analyzing nonlinear and non-conservative oscillators. Unlike classical perturbation techniques, NPA does not rely on the existence of small parameters or near-resonance conditions and therefore remains valid for strongly nonlinear regimes. The principal advantages of the NPA framework can be summarized as follows:

(i) It provides analytical representations of the system response without resorting to series expansions,
(ii) It accommodates both small and large oscillation amplitudes within a unified formulation,
(iii) It avoids truncation errors associated with perturbation-based methods, and
(iv) It allows a direct investigation of stability characteristics over a broad parameter range.

These features make NPA particularly suitable for the analysis of nonlinear oscillators with time delay, where conventional perturbation approaches may lose accuracy or fail altogether^27–35^, and El-Dib^36–41^.

Despite the extensive body of literature on Mathieu oscillators and time-delayed nonlinear systems. Diverse important limitations remain unresolved. Most existing analytical investigations rely on perturbation techniques, such as the MTSM, Lindstedt–Poincaré approaches, and averaging methods. These inherently presume weak nonlinearities, small excitation amplitudes, or near-resonance constraints. In particular, Warminski^42^ examined the nonlinear dynamics of van der Pol and Rayleigh oscillators with time delay employing the MTSM, which inherently limits the stability analysis to weakly nonlinear and perturbative regimes. Along similar lines, analytical investigations of delayed Duffing–van der Pol and parametrically excited oscillators have predominantly relied on averaging- or perturbation-based techniques, causing findings that are valid mainly near resonance conditions and for small nonlinearities^43,44^. As a consequence, the coupled influences of finite time delay and strong nonlinear damping on the stability boundaries and parametric instability zones of nonlinear Mathieu-type oscillators remain insufficiently explored. These limitations clearly motivate the development of a non-perturbative analytical framework, such as the NPA, capable of capturing strongly nonlinear dynamics under finite time-delay constraints with broader applicability.

The motivation for this study stems from the need to develop analytical tools capable of accurately describing the dynamics of nonlinear oscillators with time delay under realistic operating conditions. In many practical systems, such as vibration control devices, feedback-regulated mechanical structures, and delayed control loops, nonlinear damping and delay influences are not necessarily small and cannot be tackled as perturbations. Therefore, an analytical framework that avoids restrictive propositions on system factors is vital in capturing the true dynamic performance, including stability transitions and delay-induced modifications of parametric resonance. Addressing this challenge is crucial to bridging the gap between idealized theoretical models and real engineering systems governed by nonlinear and delayed dynamics.

The novelty of this work lies in employing the NPA to a time-delayed nonlinear Mathieu oscillator. Formerly, these systems were essentially investigated employing perturbation-based procedures, such as the MTSM adopted in^42^, which is limited to weak nonlinearities and small excitation factors. The current NPA formulation enables the derivation of analytical response and stability characteristics without relying on small-parameter or near-resonance presumption. This allows a direct analytical examination of the combined impacts of finite time delay and nonlinear damping in both van der Pol and Rayleigh oscillators. As a consequence, the study provides explicit stability characterizations in parameter regimes between the two damping models. These contributions offer a clearer and comprehensive understanding of delayed parametric excitation in strongly nonlinear oscillatory systems. Unlike former studies that treated van der Pol or Rayleigh oscillators separately or relied on perturbation-based approximations, the present work provides a unified NPA of both damping mechanisms within a time-delayed nonlinear Mathieu framework. This approach exhibits delay-induced instability regions and bifurcation structures that remain inaccessible to classical perturbation procedures. The outcomes offer practical insights for the design and control of delayed parametric systems encountered in micro-nano-electromechanical resonators, active vibration suppression, machining dynamics, and feedback-controlled engineering structures. For clarity, Table 1 summarizes the main methodological differences between the current study and selected related works.

**Table 1:** Shows a comparison between the present study and selected analytical approaches.

Study	Method	Main assumptions	Time delay	Nonlinearity regime
^9^	Perturbation-based methods	Near-resonance, small excitation	Not included	Weak
^10^	NPA-based approach	Specific damping structure	Not included	Moderate
^42^	MTSM	Small parameters, weak non-linearity, near-resonance	Included	Limited
^45^	Averaging / perturbation-based analysis	Weak nonlinearity, near-resonance, harmonic excitation	Included	Weak
Current study	NPA	No small-parameter or near-resonance presumptions	Included	Weak to strong

## Construction of the issue

**Fig. 1:**
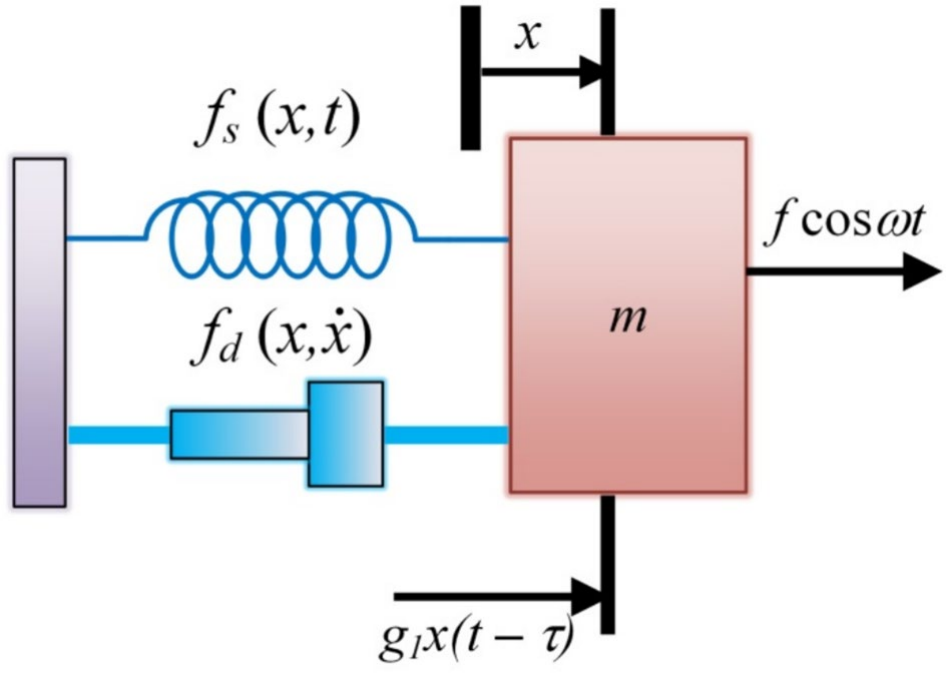
Emphasizes the nonlinear oscillator incorporating self-excitation, parametric excitation, external forcing, and time-delayed feedback.

In what follows, Fig. 1 expounds a nonlinear dynamic model representing a structurally simplified single-degree-of-freedom (1DOF) oscillator. Despite its mechanical simplicity, the model captures essential features of nonlinear oscillatory behavior arising from the combined presence of self-excitation due to nonlinear damping, parametric excitation induced by time-periodic stiffness modulation, and external harmonic forcing. Such interactions are known to generate complex dynamic responses, including resonance and stability transitions, depending on the system parameters. The model further incorporates a time-delayed feedback term characterized by a gain factor $g_{1}$ and a constant time delay $\tau$, which allows the system response to be influenced by its past states. When the delay gain is set to zero $g_{1} = 0$, the system diminishes to an uncontrolled open-loop configuration. By adjusting the gain $g_{1}$ and time delay $\tau$ magnitudes, the feedback mechanism can significantly alter the system dynamics, providing a useful framework in analysing delay-induced impacts on stability and response characteristics.

The governing dimensionless nonlinear ODE was previously given by Warminski^42^:

1 $$\ddot{x} + f_{d} (x,\,\dot{x}) + \left( {\omega_{0}^{2} - \mu \cos 2\Omega t} \right)\left( {x + \gamma x^{3} } \right) = f\cos \omega t + g_{1} x(t - \tau )$$

The mathematical model adopted in this study is taken directly from^42^, which examined the dynamics of time-delayed oscillators employing the MTSM. Retaining the same formulation enables a direct and meaningful comparison between perturbation-based and non-perturbative analytical approaches. Unlike^42^, which relies on time-scale separation and small-parameter assumptions, this work employs the NPA, allowing the analysis to remain valid for finite time delays and stronger nonlinear impacts.

In Eq. (1), $\omega_{0}$ points out the natural frequency of the oscillator, $\Omega$ reflects the frequency of parametric excitation, $\gamma$ is a factor of cubic nonlinearity. The symbol $f$ denotes the amplitude of external forcing. The symbol $\omega$ indicates the frequency of external forcing, and $g_{1}$ outlines the gain of the time-delay parameter.

Two forms of nonlinear damping are considered in this study. For the van der Pol oscillator, the damping force is expressed as:

2 $$f_{d} (x,\,\dot{x}) = ( - \alpha_{v} + \beta_{v} x^{2} )\dot{x}$$

where $f_{d}$ exhibits a nonlinear damping.

The system experiences amplitude-dependent damping characterized by negative damping at low amplitudes and positive damping at higher amplitudes, leading to self-sustained oscillations.

Alternatively, the Rayleigh oscillator is described by the nonlinear damping term.

3 $$f_{d} (x,\,\dot{x}) = ( - \alpha_{R} + \beta_{R} \dot{x}^{2} )\dot{x}$$

Where the damping depends explicitly on velocity. This form generally findings in smoother oscillatory responses and different stability characteristics compared to the van der Pol case.

These two damping mechanisms, when combined with parametric excitation, external forcing, and time-delayed feedback, give rise to distinct dynamical performances. The detailed analytical treatment and comparative investigation of these two cases are presented later in Section "Guaranteeing findings" employing the proposed NPA framework. The Rayleigh oscillator incorporates a velocity-dependent nonlinear damping mechanism, whereas the van der Pol oscillator is characterized by displacement-dependent nonlinear damping. These distinct damping formulations lead to fundamentally diverse energy exchange mechanisms within the system.

## Mathematical modelling of NPA

In this section, the NPA is systematically applied to derive analytical approximations and stability restrictions for a time delayed nonlinear Mathieu oscillator. Two representative nonlinear damping cases, namely the van der Pol and Rayleigh models, are explored to elucidate their distinct influences on the system’s dynamic response.

### Overview of the NPA methodology

**Fig. 2:**
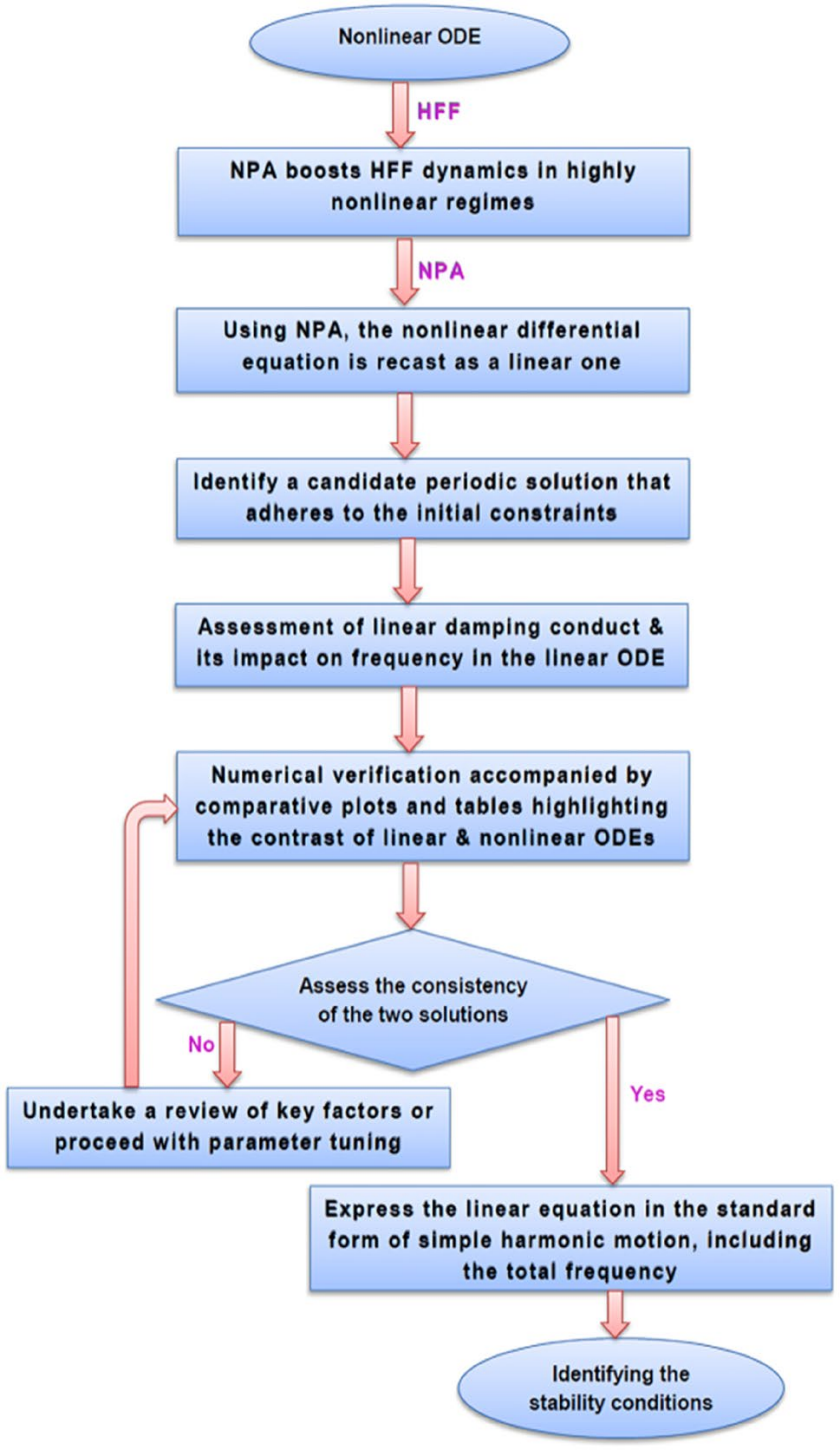
Outlines the methodology of the solution, emphasizing the HFF–NPA processes.

To enhance clarity and facilitate reproducibility, the NPA is presented through a step-by-step flowchart. This structured representation is intended to explicitly illustrate the logical sequence of the methodology and the role of each stage in the analytical process. Therefore, Fig. 2 is sketched to summarize the complete NPA procedure adopted in this investigation, highlighting how the method systematically reformulates a nonlinear ODE into an equivalent linear representation. The procedure starts by introducing an appropriate trial solution, which serves as the foundation of the analytical transformation. The resulting formulation is then assessed through numerical simulations to verify consistency with the original nonlinear model. Key findings are further organized and compared using tabulated results, allowing a transparent evaluation of accuracy and stability characteristics. This combined analytical–numerical framework ensures that the proposed NPA implementation is both systematic and verifiable, addressing the limitations of conventional perturbation-based procedures. It is important to note that the proposed framework provides a broader analytical coverage of the parameter space, which is later verified through numerical simulations in representative regimes.

From a practical and engineering perspective, these features make the proposed approach efficient and robust in predicting and controlling complex dynamical responses in nonlinear mechanical and physical systems, including vibration mitigation using nonlinear energy sinks, MEMS/NEMS devices, energy harvesting systems, and coupled oscillators, where strong nonlinearities and parameter sensitivity play a crucial role.

### Damped van der pol oscillator (Case 1)

The van der Pol oscillator is a classical model for self-excited nonlinear oscillations and has been widely used to describe systems with amplitude-dependent damping and delayed feedback. In this study, it is considered a representative case to investigate the influence of position time delay on externally excited nonlinear Mathieu-type systems. Accordingly, we have:

4 $$\ddot{x} + ( - \alpha_{v} + \beta_{v} x^{2} )\dot{x} + \left( {\omega_{0}^{2} - \mu \cos 2\Omega t} \right)\left( {x + \gamma x^{3} } \right) = f\cos \omega t + g_{1} x(t - \tau )$$

Given the initial conditions (ICs):

5 $$x\left( 0 \right) = M, \mathrm{and}\;\dot{x}\left( 0 \right) = 0.$$

A guessing (trial) solution was introduced as

6 $$x\left( t \right) = M\,\cos \Lambda t,$$

where $M$ points out the initial amplitude of vibration, and$\Lambda$ is the overall frequency.

At this stage, Eq. (4) takes the following form:

7 $$\ddot{x} + G_{1} (x,\,\dot{x}) + G_{2} (x) - \mu x\cos 2\Omega t = 0$$

where

8 $$G_{1} (x,\,\dot{x}) = ( - \alpha_{v} + \beta_{v} x^{2} )\dot{x} + \frac{1}{\Lambda }g_{1} \dot{x}\sin \Lambda \tau$$

and

9 $$G_{2} (x) = \omega_{0}^{2} \left( {x + \gamma x^{3} } \right) - \mu \gamma x^{3} \cos 2\Omega t - fx\cos \omega t - g_{1} x\cos \Lambda \tau$$

The influence of the time delay is evident in the final terms of Eqs. (8) and (9). The equivalent formula of Eq. (4) under a guess solution $\tilde{u}(t) = M\cos \Lambda \,t$ may be given as^27–34^:

10 $$\ddot{u} + \Gamma_{eqv} \dot{u} + \left( {\varpi_{eqv}^{2} - \mu \cos 2\Omega t} \right)\,u = 0$$

where

11 $$\Gamma_{eqv} = \int\limits_{0}^{2\pi /\Lambda } {\dot{\tilde{u}}\,G_{1} (\tilde{u},\,\dot{\tilde{u}})} dt/\int\limits_{0}^{2\pi /\Lambda } {\dot{\tilde{u}}^{2} \,dt}$$

and

12 $$\varpi_{eqv}^{2} = \int\limits_{0}^{2\pi /\Lambda } {\tilde{u}\,G_{2} (\tilde{u})\,} dt/\int\limits_{0}^{2\pi /\Lambda } {\tilde{u}^{2} \,dt}$$

Equation (10) reflects the standard linear damped Mathieu oscillator, which is characterized by the presence of periodic factors. Due to the presence of time-varying factors, the equation does not have an exact solution. Therefore, it typically exhibits complex dynamic performance, including parametric resonance and stability transitions. Consequently, an analytical NPA procedure must be employed again to obtain an approximate solution and incorporate the stability constraints into the system’s response.

Consequently, Eq. (10) can be reformulated as:

13 $$\ddot{u} + \Gamma_{eqv} \dot{u} + F\left( {u;\,t} \right)\,\, = 0$$

where $$F\left( {u;\,t} \right) = \left( {\varpi_{eqv}^{2} - \mu \cos 2\Omega t} \right)u$$.

A presumed form of the solution is given by:

14 $$\tilde{u}(t) = A\cos \Psi \,t$$

The equivalent form of Eq. (13) may be given as:

15 $$\ddot{u} + \Gamma_{eqv} \dot{u} + \sigma_{eqv}^{2} \,\,u = 0$$

where

16 $$\sigma_{eqv}^{2} = \int\limits_{0}^{2\pi /\Psi } {\tilde{u}\,F\left( {\tilde{u};\,t} \right)\,} dt/\int\limits_{0}^{2\pi /\Psi } {\tilde{u}^{2} \,dt}$$

At this stage, the standard normal form of Eq. (15) is employed to simplify the stability analysis and to represent the system’s conduct in a more tractable mathematical form. This transformation not only enables an approximate assessment of the stability configuration but also aids in eliminating the damping term. Accordingly, Eq. (15) can be rewritten as:

17 $$\ddot{\tilde{u}} + \Psi^{2} \tilde{u} = 0$$

where $\Psi^{2}$ denotes the new total frequency, and its detailed form is excluded to maintain conciseness in the manuscript.

Hence, the stability criteria take the following form:

18 $$\Psi^{2} > 0\;\mathrm{and}\;\Gamma_{eqv} > 0$$

### Response of damped Rayleigh oscillator (Case 2)

The Rayleigh oscillator has attracted considerable attention due to its fundamental role in modelling self-sustained oscillations driven by nonlinear damping. Consequently, we have:

19 $$\ddot{x} + ( - \alpha_{R} + \beta_{R} \dot{x}^{2} )\dot{x} + \left( {\omega_{0}^{2} - \mu \cos 2\Omega t} \right)\left( {x + \gamma x^{3} } \right) = f\cos \omega t + g_{1} x(t - \tau )$$

Under the usual ICs:

  

20 $$x\left( 0 \right) = m,\;\mathrm{and}\;\dot{x}\left( 0 \right) = 0.$$

The assumed trial (guessing) solution is given by:

21 $$x\left( t \right) = m\,\cos \chi t,$$

where $m$ outlines the initial amplitude of vibration, and $\chi$ is the total frequency.

Accordingly, Eq. (19) is expressed as:

22 $$\ddot{x} + H_{1} (\dot{x}) + H_{2} (x) - \mu x\cos 2\Omega t = 0$$

where

23 $$H_{1} (\dot{x}) = ( - \alpha_{R} + \beta_{R} \dot{x}^{2} )\dot{x} + \frac{1}{\chi }g_{1} \dot{x}\sin \chi \tau$$

and.

24 $$H_{2} (x) = \omega_{0}^{2} \left( {x + \gamma x^{3} } \right) - \mu \gamma x^{3} \cos 2\Omega t - fx\cos \omega t - g_{1} x\cos \chi \tau$$

The impact of the time delay is once again apparent in the concluding terms of Eqs. (23) and (24).

The equivalent representation of Eq. (19) based on the guessing solution $\tilde{v}(t) = m\cos \chi \,t$ is expressed as:

25 $$\ddot{v} + \eta_{eqv} \dot{v} + \left( {\delta_{eqv}^{2} - \mu \cos 2\Omega t} \right)\,v = 0$$

where

26 $$\eta_{eqv} = \int\limits_{0}^{2\pi /\chi } {\dot{\tilde{v}}\,H_{1} (\dot{\tilde{v}})} dt/\int\limits_{0}^{2\pi /\chi } {\dot{\tilde{v}}^{2} dt}$$

and

27 $$\delta_{eqv}^{2} = \int\limits_{0}^{2\pi /\chi } {\tilde{v}\,H_{2} (\tilde{v})\,} dt/\int\limits_{0}^{2\pi /\chi } {\tilde{v}^{2} \,dt}$$

Equation (25) outlines the standard linear damped Mathieu equation, distinguished by its periodic factors. The existence of these time-dependent terms renders the equation unsolvable in closed form, as it does not admit an exact analytical solution. Consequently, the system often exhibits intricate dynamic performance such as parametric resonance, instability zones, and transitions between stable and unstable regimes. Following the same non-perturbative framework adopted in Case 1, NPA is employed to derive approximate solutions and stability conditions for the Rayleigh oscillator. Accordingly, Eq. (25) can be reformulated as follows:

28 $$\ddot{v} + \eta_{eqv} \dot{v} + E\left( {v;\,t} \right)\,\, = 0$$

where $$E\left( {v;\,t} \right) = \left( {\delta_{eqv}^{2} - \mu \cos 2\Omega t} \right)v$$.

With a trial solution expressed as:

29 $$\tilde{v}(t) = B\cos \Phi \,t$$

Equation (28) can be reformulated in the following equivalent form.

30 $$\ddot{v} + \eta_{eqv} \dot{v} + \Sigma_{eqv}^{2} \,\,v = 0$$

where

31 $$\Sigma_{eqv}^{2} = \int\limits_{0}^{2\pi /\Phi } {\tilde{v}\,E\left( {\tilde{v};\,t} \right)\,} dt/\int\limits_{0}^{2\pi /\Phi } {\tilde{v}^{2} \,dt}$$

At this point, Eq. (30) is expressed in its standard normal form to simplify stability analysis and provide a more convenient representation of the system’s performance. This reformulation not only facilitates an easier evaluation of the system’s stability but also aids in excluding the damping term, isolating primary parametric influences. Thereby, Eq. (30) can be rewritten as follows:

32 $$\ddot{\tilde{v}} + \Phi^{2} \tilde{v} = 0$$

where $\Phi$ corresponds to total frequency and has been excluded here to streamline the presentation.

Therefore, the constraints for stability can be written as:

  

33 $$\Phi^{2} > 0\;\mathrm{and}\;\eta_{wqv} > 0$$

## Guaranteeing findings

This section verifies the analytical outcomes employing numerical simulations, Tabular & graphical comparisons, and comparisons with existing studies, confirming the accuracy and robustness of the proposed non-perturbative framework.

### Comparative validation via tabulated data

A quantitative validation of the proposed NPA is presented in Tables 2 and 3 through direct comparison with numerical solutions obtained using the NDSolve routine. The comparison is performed for an excited nonlinear Mathieu oscillator with time delay under two distinct damping mechanisms. Table 2 corresponds to the van der Pol oscillator case, while Table 3 addresses the Rayleigh oscillator case.

**Table 2:** Displays a comparison of convergence between the NS and the NPA approximation for the van der Pol oscillator.

Time	Numerical	Equivalent	Absolute error	Relative absolute error (%)
0	0.05	0.05	0.00	0.00
3	0.0536239	0.0536365	0.0000125888	0.0234761
6	0.0448435	0.0448559	0.0000123752	0.0275964
9	0.0205761	0.0205683	0.00000782931	0.0380505
12	− 0.0195534	− 0.0196098	0.0000563448	0.288158
15	− 0.0719409	− 0.0720821	0.000141196	0.196267
18	− 0.128219	− 0.128487	0.000267708	0.208789
21	− 0.175379	− 0.175814	0.000434724	0.247877
24	− 0.197151	− 0.197781	0.000629906	0.319505
27	− 0.176845	− 0.177669	0.000824	0.465944
30	− 0.101526	− 0.102492	0.000965797	0.951282

**Table 3:** Displays a comparison of convergence between the NS and the NPA approximation for the Rayleigh oscillator.

Time	Numerical	Equivalent	Absolute error	Relative absolute error (%)
0	0.05	0.05	0.00	0.00
3	− 0.0659587	− 0.0659892	0.0000305238	0.0462771
6	0.0578297	0.0578452	0.0000154937	0.0267919
9	− 0.0314773	− 0.0315079	0.0000305876	0.0971735
12	− 0.0141944	− 0.0142295	0.000035139	0.247556
15	0.0760596	0.076179	0.00011941	0.156995
18	− 0.145332	− 0.145661	0.000329119	0.22646
21	0.20777	0.208326	0.000556252	0.267725
24	− 0.244552	− 0.245333	0.00078086	0.319302
27	0.235549	0.236183	0.000634595	0.269411
30	− 0.163524	− 0.163194	0.000329227	0.201333

In both Tables, the NSs of the governing Eqs. (4) and (19) are compared with the corresponding analytical approximations derived via the NPA, given by Eqs. (15) and (30), respectively. The absolute and relative errors are reported at selected time instants to assess the accuracy of the analytical formulation. The results demonstrate an excellent agreement between the numerical and analytical solutions over the considered time interval. The consistently small error magnitudes confirm that the NPA accurately captures the system response in the presence of nonlinear damping and finite time delay. This agreement validates the reliability of the proposed framework for both self-excited (van der Pol) and velocity-dependent (Rayleigh) damping cases and ensures its suitability for analyzing time-delayed nonlinear oscillatory systems.

 Table 2: $$A = 0.05,$$ $$g_{1} = 0.002,$$ $$f = 0.005,$$ $$\mu = 0.001,$$ $$\sigma = 0.005,$$ $$\omega _{0} = 2.24,$$ $$\omega = 0.001,$$ $$\gamma = 0.005,$$ $$\tau = 0.001,$$ $$\alpha_{v} = 0.12,$$ and $$\beta_{v} = 0.001.$$ Table 3: $$B = 0.05,$$$$g_{1} = 0.0002,$$$$f = 0.0001,$$$$\mu = 0.0001,$$$$\sigma = 0.001,$$$$\omega _{0} = 2.24,$$$$\omega = 0.0001,$$$$\gamma = 0.0005,$$$$\tau = 0.001,$$ $$\alpha_{R} = 0.12,$$ and $$\beta_{R} = 0.002.$$

The close agreement between the NS and NPA simulations ensures the accuracy and robustness of the proposed analytical framework in capturing the nonlinear dynamics of the time-delayed Mathieu oscillator.

### Visual validation via schematic diagrams

**Fig. 3:**
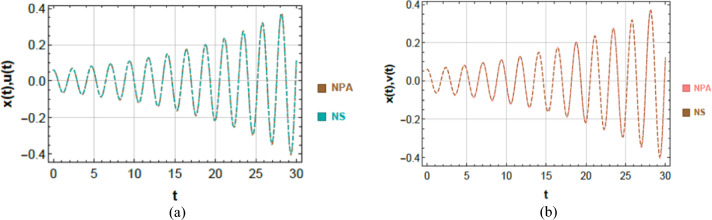
(**a**) Comparison between the NS of Eq. (4) and the NPA of Eq. (15) for the van der Pol oscillator. (**b**) Comparison between the NS of Eq. (19) and the NPA of Eq. (30) for the Rayleigh oscillator.

To further validate the analytical predictions obtained via the non-perturbative approach, a visual comparison with numerical solutions is presented in Fig. 3a and b. These figures illustrate the time-domain responses computed using the numerical solver (NDSolve) and the corresponding approximate solutions derived via NPA. The comparison is conducted for the same parameter sets considered in the tabulated validation, ensuring consistency across validation methods.

Figure 3a corresponds to the van der Pol oscillator case, which is characterized by self-excited nonlinear oscillations under time-delayed parametric excitation. Figure 3b expounds the Rayleigh oscillator case, where the dynamics are governed by velocity-dependent nonlinear damping. In both cases, the NPA outcomes given by Eqs. (15) and (30), closely follow the numerical solutions of Eqs. (4) and (19), respectively. The assigned quantities to these dimensionless factors within the framework are provided below:$B = 0.06,\,\,A = 0.06,\,g_{1} = 0.0002,\,f = 0.005,\mu = 0.0001,$ $\gamma = 0.0005,$ $\omega_{0} = 2.63,$ $\sigma = 0.005,\,$ $\omega = {0}.0001,\,$ $\tau = 0.001,\,\alpha_{v} = 0.13;\,\beta_{v} = 0.052,\,\alpha_{R} = 0.13,$ and $\beta_{R} = 0.002.$

The excellent overlap between the numerical and analytical curves confirms the ability of the NPA to accurately reproduce the nonlinear dynamic response of the time-delayed Mathieu oscillator. For the van der Pol case, the maximum deviation between the two solutions is approximately 0.0117517, while a similar deviation of about 0.0117331 is noticed for the Rayleigh case. These small discrepancies demonstrate the robustness and reliability of the proposed analytical framework in capturing the impact of nonlinear damping and finite time delay.

The visual validation outcomes indicate that the form of nonlinear damping plays a decisive role in shaping the system response. In particular, the van der Pol oscillator exhibits relatively larger steady-state amplitudes due to its amplitude-dependent damping mechanism, whereas the Rayleigh oscillator displays smoother responses with comparatively lower amplitudes under similar parameter constraints. These qualitative differences are consistently captured by both the numerical simulations and the NPA-based analytical solutions, thereby reinforcing the robustness and reliability of the proposed framework.

### Comparisons with previous literature

A closely related investigation was recorded by Wang and Li^45^, who examined the dynamical properties of a Duffing–van der Pol oscillator under combined external and parametric excitations with time-delayed feedback employing a perturbation-based procedure. Their study demonstrated that the interaction between nonlinear damping, excitation, and delay can trigger transitions from periodic to chaotic responses through mechanisms such as period-doubling and intermittency. The chaotic features found in their bifurcation schemes and PMs are qualitatively consistent with those identified in the current work. However, their analysis was confined to weakly nonlinear regimes and near-resonance constraints.

A similar limitation exists in^42^, where van der Pol and Rayleigh oscillators with time delay were analyzed employing the MTSM, restricting the validity of the findings to perturbative presumption and preventing the derivation of explicit stability boundaries of finite delay. In contrast, the present work employs the NPA, which allows the analytical construction of stability boundaries without relying on small parameters or near-resonance constraints. As a consequence, delay-induced stability and instability zones that are inaccessible to perturbation-based or purely numerical approaches are explicitly revealed. Over and above, the analysis of transient chaotic phases highlights the essential role of delay-induced memory impacts in shaping the global phase-space structure, thereby extending and refining the dynamical insights recorded by^42,45^. Finally, these comparisons demonstrate that the current study provides a more comprehensive and physically representative characterization of delayed nonlinear Mathieu oscillators, offering improved analytical predictability for complex delayed parametric systems in practical engineering implementations.

## Interpretation and physical insights

In what follows, This section provides a detailed physical interpretation of the analytical and numerical outcomes obtained for the two investigated systems. The discussion focuses on elucidating the impact of key system parameters on the dynamical behavior through time histories, phase portraits, stability schematics, and polar representations. Particular attention is given to clarifying how time delay, nonlinear damping, and parametric excitation collectively shape the system response, stability limits, and energy transfer mechanisms. This Section bridges the gap between the mathematical formulation and the physical performance of the van der Pol and Rayleigh oscillators, offering clear insights into the underlying nonlinear dynamics.

### Insight into the damped van der pol oscillator

#### Time history analysis for case 1

Time history analysis provides a direct temporal description of the system response and constitutes a fundamental tool for interpreting the evolution of nonlinear oscillations under delayed feedback. By exploring the displacement response as a function of time, this approach enables the identification of transient conduct, amplitude growth or decay, and the onset of instability or sustained oscillations. In this study, time history analysis is particularly valuable for clarifying how the interplay between nonlinear damping, parametric excitation, and time delay governs the dynamic performance of the excited nonlinear Mathieu oscillator. For the van der Pol oscillator, time histories offer clear physical insight into the mechanism of self-excited oscillations and how delayed feedback modifies their amplitude, persistence, and stability. In particular, the presence of a time delay alters the effective energy balance of the system, potentially enhancing oscillatory movement or delaying the decay of transients, depending on the chosen factors’ magnitudes.

**Fig. 4:**
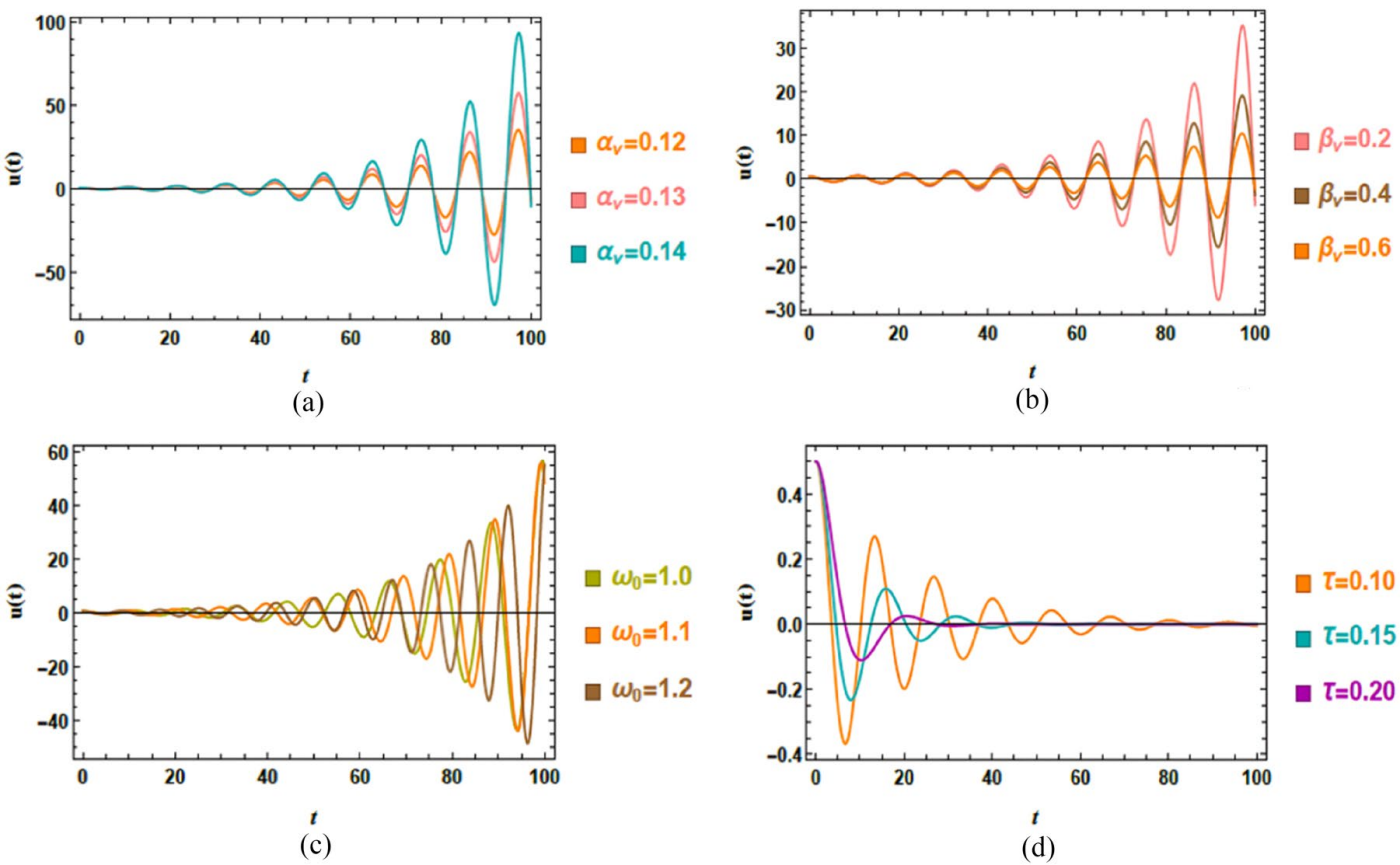
(**a**) Outlines the effect of $\alpha_{v}$ on $u\left( t \right)$. (**b**) Clarifies the conduct of $\beta_{v}$ on $u\left( t \right)$. (**c**) Shows the impact of $\omega_{0}$ on $u\left( t \right)$. (**d**) Depicts the influence of $\tau$ on $u\left( t \right)$.

Accordingly, Figs. 4a–d demonstrate the impact of key system factors on the displacement response $u\left( t \right)$. The investigated factors include the linear damping factor $\alpha_{v}$ and the nonlinear damping factor $\beta_{v}$, which jointly regulate energy dissipation and self-excitation; the natural frequency $\omega_{0}$, which controls resonance conditions; and the time delay $\tau$, which introduces memory impacts into the system dynamics. These factors are chosen to highlight their distinct and combined roles in shaping the temporal evolution, stability, and amplitude modulation of the van der Pol–type response. The specific dimensionless magnitudes adopted in the simulations are listed below.

$$A = 0.5,{\mkern 1mu} {\mkern 1mu} {\mkern 1mu} g_{1} = 2.0002,{\mkern 1mu} \mu = 0.0001,\;f = 0.27,\mu = 0.0001,\sigma = 0.0001,{\mkern 1mu} {\mkern 1mu} \omega _{0} = 1,\omega = 3,\gamma = 0.00015,\;\tau = 0.01,{\mkern 1mu} \alpha _{v} = 0.12,\;{\mathrm{and}}\;\beta _{v} = 0.2.$$             

Figure 4a explores the performance of the linear damping factor $\alpha_{v}$ on the dynamic response $u\left( t \right)$ by comparing three representative values. As $\alpha_{v}$​ escalates from 0.12 to 0.14, the oscillation amplitude systematically decreases, reflecting stronger damping and more efficient energy dissipation. At the lowest damping $\alpha_{v} = 0.12$, the system exhibits more sustained and pronounced oscillations, particularly in later stages, indicating longer energy retention and slower convergence to equilibrium. In contrast, higher damping $\alpha_{v} = 0.14$ leads to significantly reduced amplitudes, demonstrating faster energy loss and quicker stabilization. This performance is consistent with damped oscillatory systems, where escalated damping suppresses excessive motion and accelerates convergence. These insights can inform the design of vibration control strategies in engineering systems with delayed feedback, such as adaptive dampers, soft robotics, or precision mechanical oscillators.

Figure 4b depicts the role of the damping factor $\beta_{v}$ on the time-dependent response $u\left( t \right)$, evaluated for three distinct amounts. As $\beta_{v}$ elevates from 0.2 to 0.6, the oscillation amplitude decreases significantly, especially in the later stages of the response, indicating more efficient energy dissipation. At the lowest value $\beta_{v} = 0.2$, the system exhibits a marked growth in amplitude over time, reflecting limited damping and a stronger tendency toward sustained oscillatory motion. In contrast, higher values of $\beta_{v}$​ produce pronounced attenuation of oscillations, demonstrating enhanced damping performance and improved system stability. Physiologically, this shows the amplitude-dependent role of nonlinear damping in regulating energy accumulation, while practically, these results highlight how tuning $\beta_{v}$​ can be used to control oscillation amplitudes in delayed feedback systems, which is relevant for applications in adaptive vibration control and precision mechanical oscillators.

Fluctuations of natural frequency $\omega_{0}$ affect dynamic response $u\left( t \right)$, as depicted in Fig. 4c. As $\omega_{0}$ increases from 1 to 1.2, the system exhibits significantly amplified oscillations, indicating a strong sensitivity to frequency modulation. This conduct is characteristic of a parametric resonance mechanism, where the natural frequency becomes favorably aligned with the internal or external periodic excitation, causing pronounced energy transfer into the system. The progressive growth of oscillation amplitude at higher amounts of $\omega_{0}$​ suggests a transition from a stable to an unstable regime, which is particularly evident near the end of the simulation interval, where oscillations increase rapidly. From a physical perspective, this highlights the critical role of frequency tuning in delayed nonlinear systems, as small deviations in $\omega_{0}$​ can trigger instability and dramatically alter the system response, an influence that is essential for both resonance avoidance and controlled excitation in practical implementations.

Figure 4d demonstrates the impact of varying time delay $\tau$ on the dynamic response $u\left( t \right)$. As the time delay enlarges, the system exhibits more sustained oscillations accompanied by a noticeably slower decay rate, indicating a reduction in effective damping and a loss of stability. Physically, the introduction of a time delay generates a phase mismatch between the current state and the delayed feedback, which alters the instantaneous energy balance. This delay-induced mechanism causes prolonged transients and, for sufficiently large $\tau$, may promote the onset of instability or quasi-sustained oscillatory performance. These results demonstrate that time delay acts as a critical control parameter, capable of shifting stability boundaries and generating dynamic regimes that do not exist in the delay-free system, which is of direct relevance to the design and control of engineering systems with delayed feedback.

### Phase plane dynamic analysis for case 1

Phase plane analysis is a powerful qualitative tool for examining the dynamic performance of nonlinear ordinary differential equations by representing system trajectories in a two-dimensional state space, typically defined by the displacement $\tilde{u}\left( t \right)$ and the velocity $\dot{\tilde{u}}\left( t \right)$. Unlike time-history analysis, which emphasizes temporal evolution, phase plane representations provide direct insight into equilibrium states, trajectory convergence or divergence, and global stability characteristics. In the context of the excited nonlinear Mathieu oscillator with time delay, and particularly for the van der Pol oscillator, this approach is especially effective in revealing how time-delayed feedback modifies self-excited oscillations and alters stability properties.

**Fig. 5:**
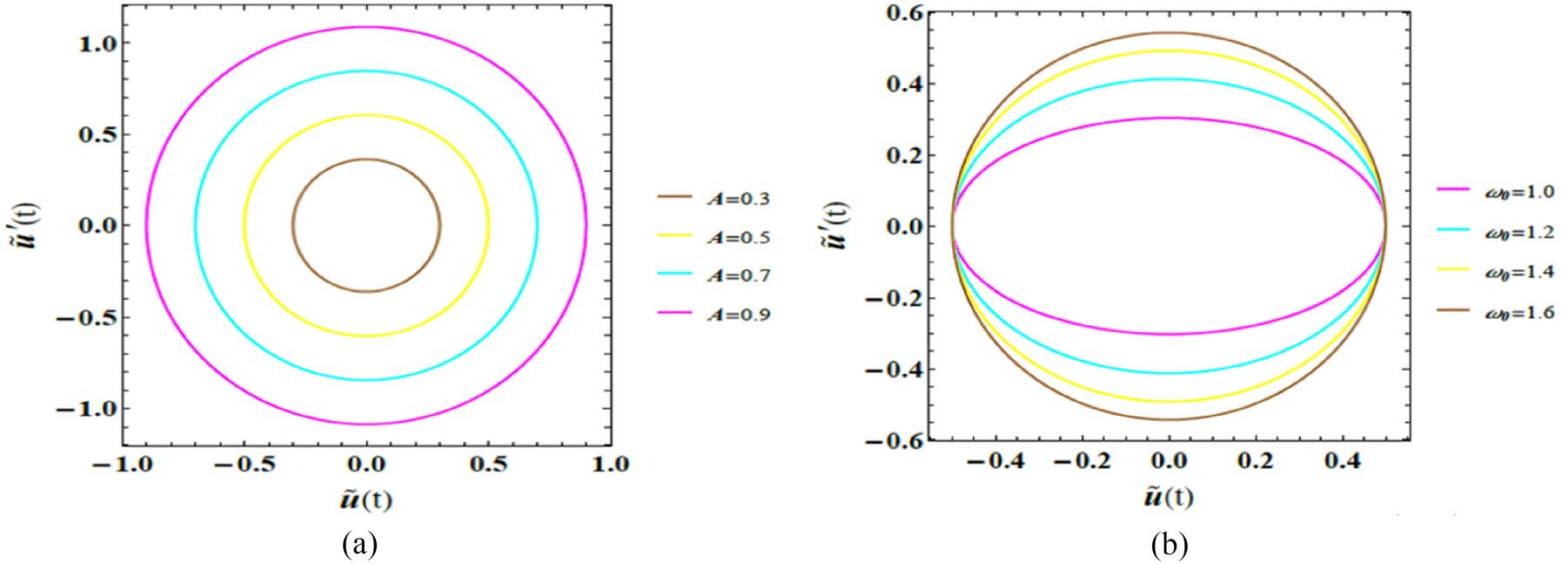
(**a**) Shows the conduct of $A$ on the $\left( {\tilde{u},\,\dot{\tilde{u}}} \right)$ plane. (**b**) Reflects the impact of $\omega_{0}$ on the $\left( {\tilde{u},\,\dot{\tilde{u}}} \right)$ plane.

Closed or bounded trajectories in the phase plane indicate periodic or sustained oscillations, whereas spiraling trajectories toward or away from equilibrium reflect damping dominance or instability, respectively. For nonlinear systems, where analytical solutions are generally unavailable, phase portraits offer a clear visualization of the combined influence of system parameters and ICs on long-term performance, enabling the identification of limit cycles, transition to instability, and delay-induced bifurcation features. Figures 5a and b exhibit representative phase trajectories in the $\left( {\tilde{u},\,\dot{\tilde{u}}} \right)$ plane, demonstrating the dynamic response of the van der Pol–type system. The dimensionless factor magnitudes utilized here are consistent with those adopted in the time-history analysis.

Figure 5a highlights the performance of amplitude $A$ on the phase plane $\left( {\tilde{u},\,\dot{\tilde{u}}} \right)$. As the amplitude $A$ elevates from 3 to 9, the closed loops expand noticeably, indicating a significant growth in both displacement and velocity magnitudes. This enlargement of the phase trajectories reflects an increase in the system’s total energy, since higher amplitudes correspond to greater mechanical and kinetic energy stored in the oscillatory motion. Despite this growth, the persistence of closed trajectories confirms the existence of stable limit cycles, characteristic of self-excited van der Pol–type dynamics. From a physical standpoint, these outcomes demonstrate that amplitude modulation governs the energy level of oscillations without destroying periodicity, highlighting the robustness of the nonlinear limit-cycle performance.

Figure 5b, the phase plane $\left( {\tilde{u},\,\dot{\tilde{u}}} \right)$ shows how the natural frequency $\omega_{0}$ shapes the system’s movement. As $\omega_{0}$ enhances from 1 to 1.6, the phase portraits evolve into more elongated and oval-shaped closed loops, with trajectories stretching along the displacement axis while maintaining smooth, bounded paths. This deformation reflects a higher oscillation rate and a faster dynamic response, as escalating $\omega_{0}$​ enables the system to complete more oscillation cycles within a given time interval. Physically, the elongation of the phase trajectories indicates enhanced energy levels and sharper transitions between displacement and velocity, while the persistence of closed orbits confirms the maintenance of periodic motion.

Stability Characteristics of Case 1                                                                                                                                                                                                                   To obtain a deeper understanding of the system’s global stability characteristics, it is essential to examine how variations in key system parameters influence the overall dynamic response. This objective is achieved by analyzing the stability restrictions that were shown in the derived in Eqs. (18) and (33). These conditions show that the system dynamics are governed by the sign of the effective squared frequency. When this quantity remains positive, the oscillator exhibits bounded periodic motion, indicating that the combined impacts of nonlinear damping, parametric excitation, and time delay are insufficient to destabilize the response.

From the physical view, this corresponds to a balance between energy input and dissipation, where perturbations are absorbed, and the system settles into stable oscillations. In contrast, a negative effective squared frequency signifies the onset of instability, where delay-induced feedback and nonlinear impacts inject energy faster than it can be dissipated, leading to exponential growth of disturbances. This mechanism is analogous to well-known instability phenomena such as parametric resonance and buckling in mechanical systems. Consequently, these stability criteria provide a clear physical explanation for the stable and unstable areas noticed in the numerical bifurcation diagrams, PMs, and LE analyses for Case 1, thereby reinforcing the consistency between the analytical predictions and the computed dynamical responses.

Employing NPA, the stability criteria can be derived in a direct and systematic manner, without relying on conventional perturbation propositions. In the current analysis, NPA is applied in two successive steps: the first step eliminates the circular trigonometric terms arising from the oscillatory response, while the second step removes the nonlinear contributions in the governing ODE. Through this procedure, the stability constraints naturally emerge from the intrinsic limitations of the NPA formulation, causing explicit and tractable stability criteria.

**Fig. 6:**
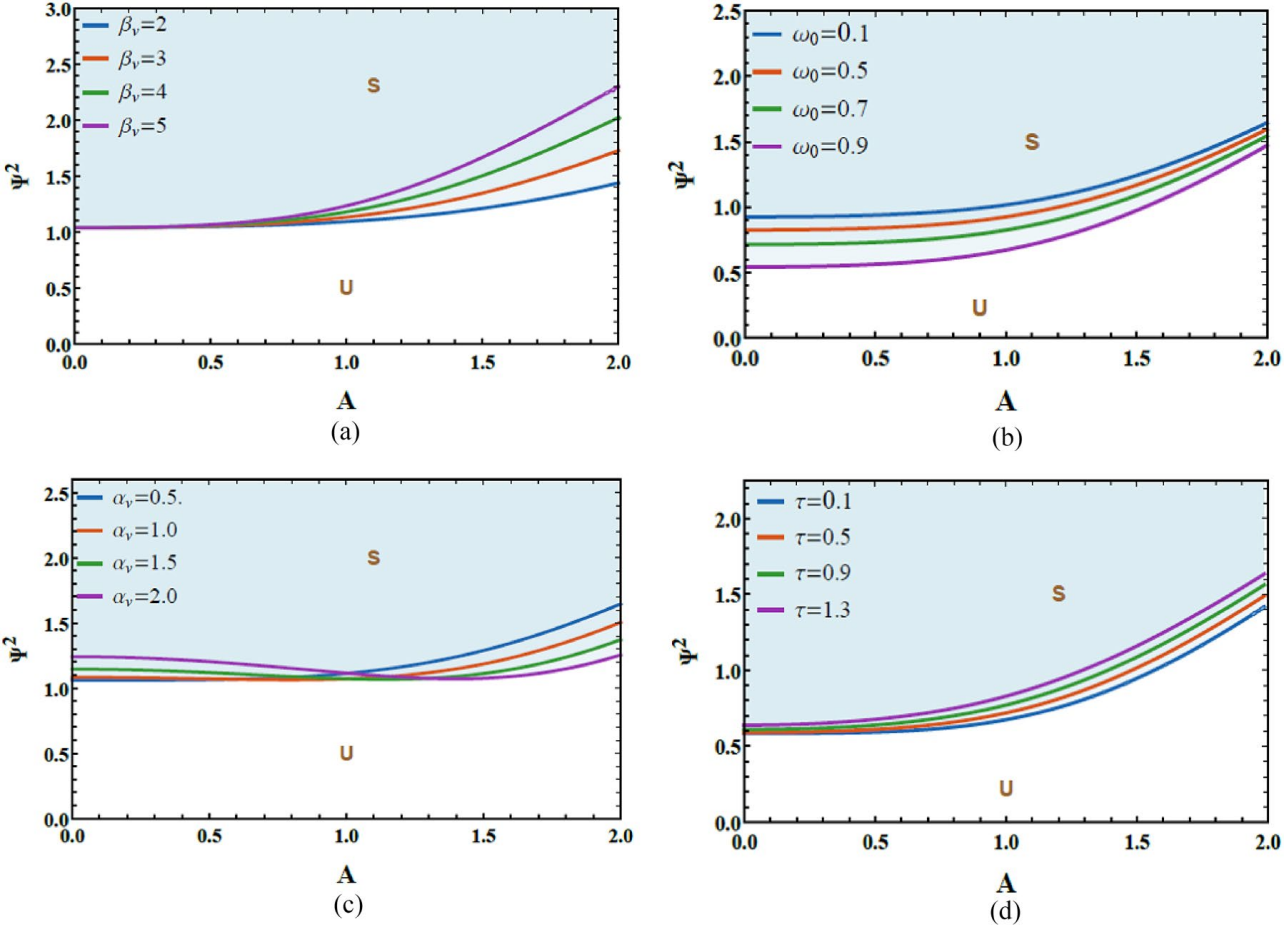
(**a**) Reflects the impact of $\beta_{v}$ on the stability graph. (**b**) Shows the conduct of $\omega_{0}$ on the stability diagram. (**c**) Describes the conduct of $\alpha_{v}$ on the stability scheme. (**d**) Exposes the performance of $\tau$ on the stability profile.

Based on these criteria (18), Figs. 6a–d examine the variation of the total frequency $\Psi^{2}$ versus the initial amplitude $A$ to assess the system’s stability allocation. These diagrams are constructed using the MS (version 12) and clearly distinguish between stable and unstable regimes under diverse physical constraints. In the diagrams, shaded areas above each curve denote stable convergence (S), whereas unshaded zones below represent unstable performance (U). This graphical representation provides a clear and physically interpretable criterion for identifying the influence of initial excitation on system stability. The dimensionless factors utilized in these simulations are listed as follows:

$$g_{1} = 0.5,\,\,f = 0.5,\,\mu = 2.0,\,\,\sigma = 0.001,\,\omega_{0} = 1,$$ $$\omega = 3.0,\,$$ $$\tau = 0.5,$$ $$\gamma = 0.01,$$ $$\,\alpha_{v} = 0.02,$$ and $$\beta_{v} = 3.$$

Figure 6a is graphed to explain how the system’s performance is influenced by fluctuations in the damping factor $\beta_{v}$. It is explored from Fig. 5a that the stable region in the stability diagram diminished with the escalation in $\beta_{v}$ from 2 to 5. From a physical standpoint, this conduct arises from the amplitude-dependent nature of the van der Pol–type damping, which governs the system’s energy balance in a nonlinear manner. At low oscillation amplitudes, the damping term acts dissipatively, extracting energy from the system and enlarging stability. However, as the amplitude escalates, the same nonlinear damping mechanism can effectively re-inject energy into the system, supporting the development of self-excited oscillations. When coupled with periodic excitation and time-delayed feedback, this nonlinear energy exchange amplifies destabilizing effects, resulting in a contraction of stable zones and an expansion of unstable areas.

The variation in the natural frequency $\omega_{0}$ on the stability schematic is explored in Fig. 6b. It is found that as natural frequency $\omega_{0}$ elevates from 0.1 to 0.9, the stable zones escalate. Accordingly, the natural frequency $\omega_{0}$ plays a pivotal role in stabilizing the system’s response. From a physical perspective, escalating the natural frequency shifts the system away from critical resonance constraints associated with the parametric excitation and delayed feedback, thereby reducing the efficiency of energy transfer into unstable modes. A higher $\omega_{0}$​ also enhances the effective stiffness of the oscillator, which limits large-amplitude responses and suppresses the growth of self-excited oscillations. Consequently, perturbations are more likely to remain bounded, and the system exhibits improved robustness against instability.

Figure 6c is portrayed to expound the influence of variations in the linear damping factor $\alpha_{v}$ on the system’s stability characteristics. It is evident from Fig. 6c that $\alpha_{v}$ plays a dual role in shaping the stability profile. Along the interval $\left[ {0,\,\,1} \right]$, escalating $\alpha_{v}$ expands the unstable zones, indicating a destabilizing influence on the system response. In contrast, for the interval $\left[ {1.1,\,\,\,2} \right]$, further enhancements $\alpha_{v}$ cause a contraction of the instability domains, thereby enhancing system stability. From a physical visualization, this conduct reflects the competing influence of linear damping on energy dissipation and nonlinear dynamic interactions. At lower damping levels, the damping term may be insufficient to counteract the energy injected through parametric excitation and nonlinear impacts, allowing oscillatory instabilities to grow. However, beyond a critical threshold, linear damping becomes dominant, effectively dissipating energy and suppressing the growth of perturbations, which stabilizes the oscillatory motion.

The variation in the time delay $\tau$ on the stability schematic is explored in Fig. 6d. It is found that as the time delay $\tau$ elevates, the stable regions in the stability diagram progressively contract, indicating a pronounced destabilizing performance on the system response. Accordingly, the time delay plays a pivotal role in diminishing the stability margins of the nonlinear Mathieu oscillator. In physical terms, time delay introduces memory impacts that cause the feedback force to act out of phase with the instantaneous system motion. This phase mismatch can effectively inject energy into the system instead of dissipating it, thereby amplifying oscillations and promoting instability. As the delay escalates, the difference between the system state and the delayed feedback becomes more significant, leading to enhanced parametric excitation and the onset of instability. This mechanism explains the noted shrinkage of stable regions and highlights the vital role of delay-induced feedback in governing the stability of time-delayed nonlinear oscillatory systems.

### Polar representation of case 1

**Fig. 7:**
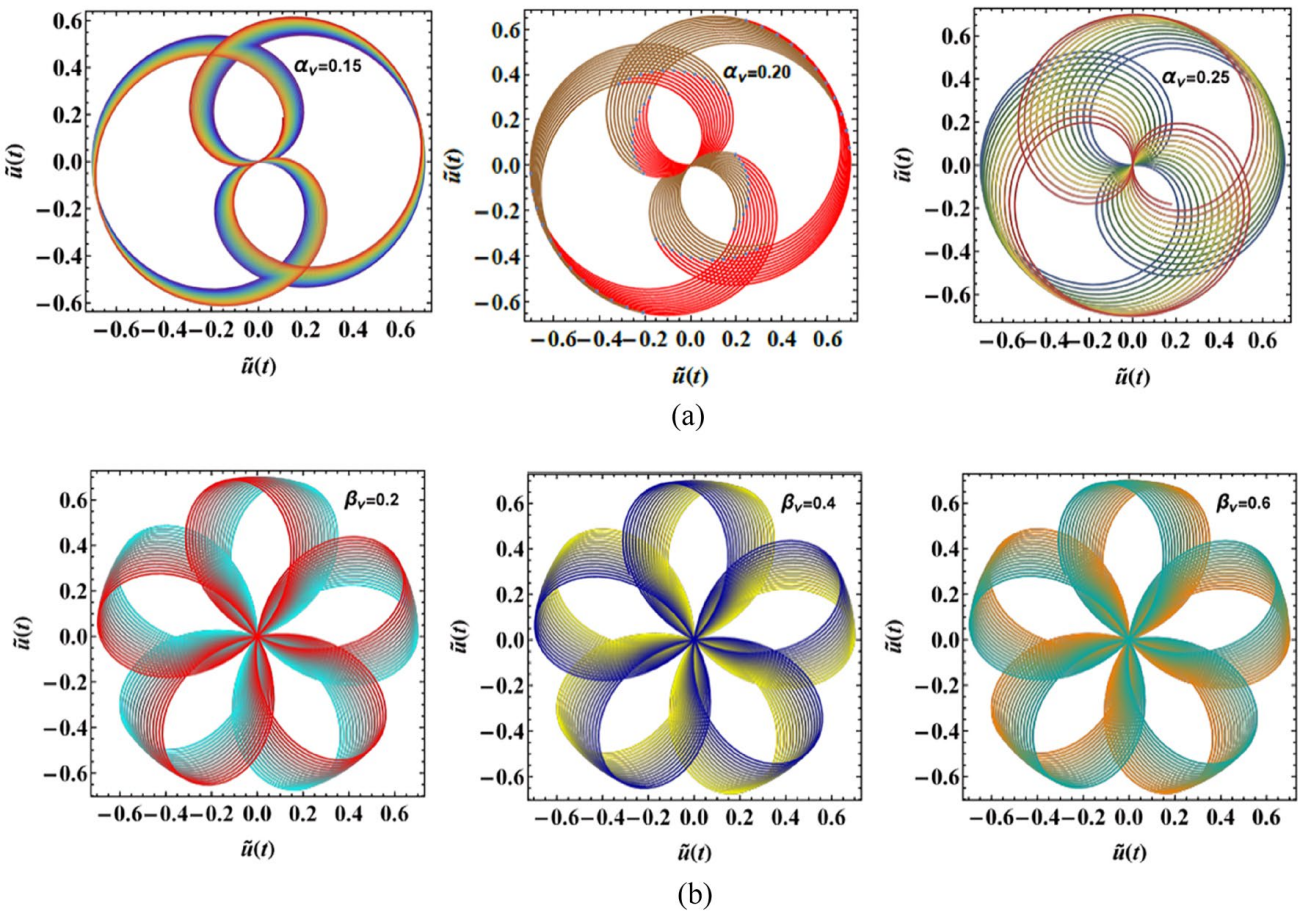
(**a**) Exhibits the PolarPlot schematic of $\hat{u}(t)$ vs. $\hat{u}(t)$ affected by $\alpha_{v}$. (**b**) Shows the PolarPlot schematic of $\hat{u}(t)$ vs. $\hat{u}(t)$ influenced by $\beta_{v}$.

The PolarPlots schematics are shown in Fig. 7a and b to offer a visual insight into the system’s periodic performance under varying $\hat{u}(t)$, as described in Eq. (14). These plots reflect how distinct factors, such as nonlinear damping factors $\alpha_{v}$, and $\beta_{v}$, shape the rotational dynamics of the oscillator. Relying on the selected parameter amounts, the trajectories form closed loops or spiral patterns that either contract or expand, reflecting the competition between energy dissipation and energy injection within the system. Variations in these factors cause noticeable changes in both the oscillation amplitude and angular progression, indicating shifts in the effective balance between nonlinear damping, delayed feedback, and excitation.

### Time history analysis of case 2

**Fig. 8:**
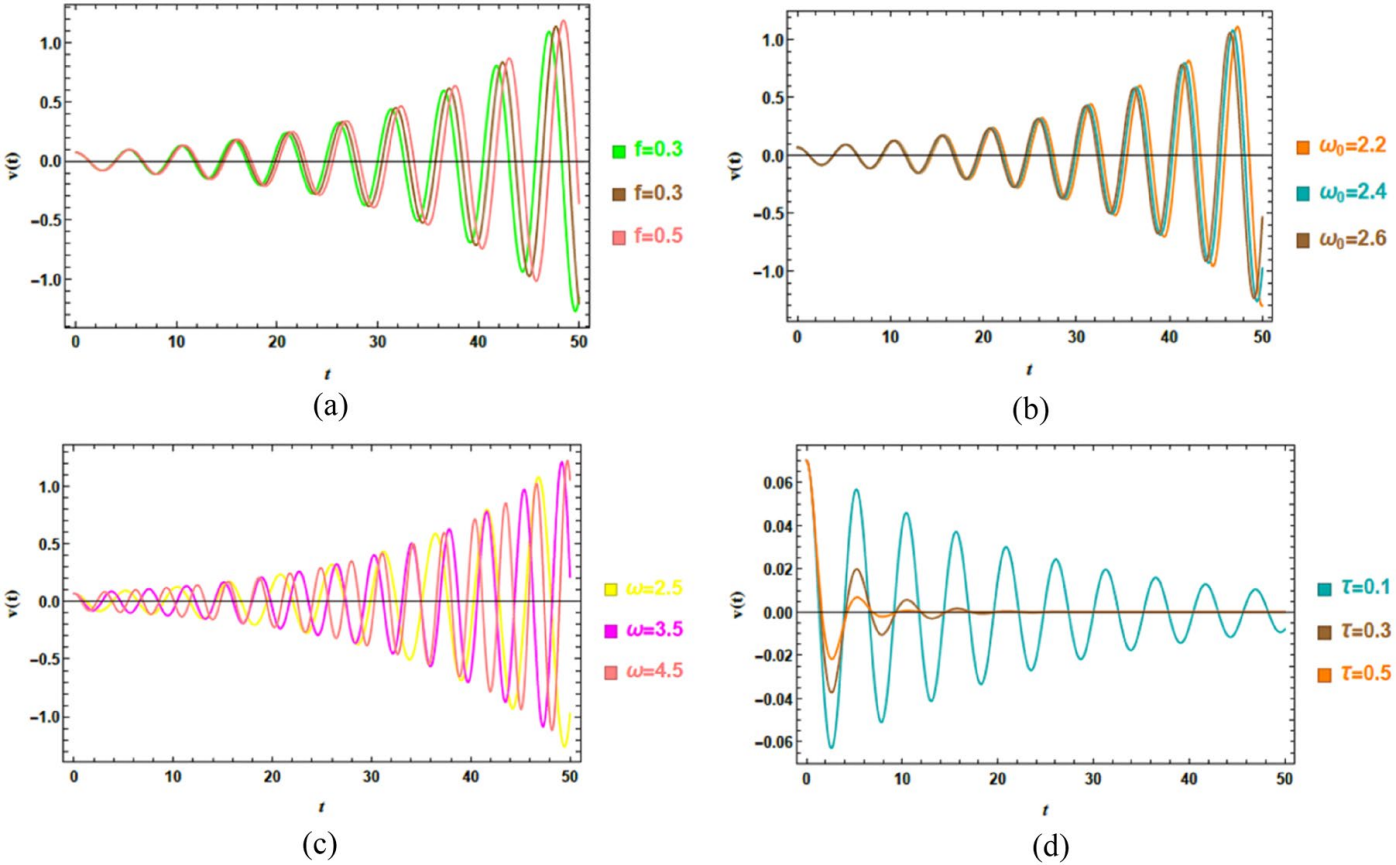
(**a**) Shows the conduct of $f$ on $v\left( t \right)$. (**b**) Expounds the influence of $\omega_{0}$ on. (**c**) Elucidates the influence of $\omega$ on $v\left( t \right)$. (**d**) Reflects the impact of $\tau$ on $v\left( t \right)$.

Figure 8a–d explore the influence of several key parameters on the time-dependent displacement response $$v\left( t \right)$$ of the excited nonlinear Mathieu oscillator with time delay, considering the Rayleigh oscillator case. Physiologically, these time history plots provide direct insight into how external excitation, intrinsic system properties, and delayed feedback jointly govern energy exchange, stability, and oscillatory persistence in nonlinear systems. The specific dimensionless measures employed in these schemes are selected to be as follows: $$B = 0.07,$$ $$g_{1} = 2.0002,$$$$f = 0.27,$$$$\mu = 0.0001,$$$$\sigma = 0.001,$$ $$\omega_{0} = 2.4,\,\,\gamma = 0.00015,\,$$ $$\tau = 0.001,\,\,\alpha_{R} = 0.12,$$ $$\omega = 2.5,$$ and $$\beta_{R} = 0.2.$$

Figure 8a demonstrates the conduct of escalating the amplitude of the external force $f$ on the dynamic response $v\left( t \right)$. From a physical view, increasing the force amplitude corresponds to injecting more energy into the oscillator. As $f$ enhances, the system exhibits stronger and more energetic oscillations, indicating that the supplied energy exceeds the dissipated energy due to damping. This enhanced energy input amplifies the displacement response and sustains oscillatory motion over longer time intervals. In practical terms, this conduct reflects how externally driven mechanical or electromechanical systems may experience amplified vibrations when subjected to stronger periodic forcing.

The impact of natural frequency $\omega_{0}$ on $v\left( t \right)$ is depicted in Fig. 8b. From a physical perspective, increasing $\omega_{0}$ shifts the system closer to resonance conditions with the parametric excitation and delayed feedback. As $\omega_{0}$ enlarges from 2.2 to 2.6, oscillations become stronger and more persistent, signaling a reduced ability of the system to suppress vibratory energy. This performance is characteristic of parametric resonance, where tuning the natural frequency to specific excitation relationships causes energy accumulation rather than dissipation. Physically, this indicates a transition toward instability, as small perturbations are increasingly amplified over time.

Figure 8c exhibits variation in the frequency of external forcing $\omega$ on the dynamic response $v\left( t \right)$. As $\omega$ enriches from 2.5 to 4.5, the system undergoes a dramatic escalation in oscillation amplitude. From a physical visualization, enhancing $\omega$ modifies the timing of energy injection into the system. When the excitation frequency approaches a critical interaction with the system’s natural and delay-induced frequencies, energy is transferred more efficiently through parametric resonance. Consequently, oscillations amplify and become increasingly irregular, signalling a loss of stability and a transition from stable periodic movement to unstable dynamic performance.

The change in time delay $\tau$ on the dynamic response $v\left( t \right)$ is examined in Fig. 8d. As time delay escalates, the system exhibits more sustained oscillations with noticeably slower decay. From a physical view, time delay iintroduces a phase mismatch between the feedback forces and the system motion. As the time delay $\tau$ elevates, the feedback forces act increasingly out of phase with the displacement and velocity, thereby reducing the effective damping action. Consequently, oscillations decay more slowly and may persist for longer durations, indicating that the system retains energy rather than dissipating it efficiently. This energy accumulation leads to a reduction in dynamic stability and promotes instability. In practical implementations, such delay-induced impacts commonly arise in control systems, mechanical actuators, and engineering structures involving sensing, signal transmission, or actuation delays, where excessive time delay can severely compromise system stability.

### Phase portrait analysis of case 2

**Fig. 9:**
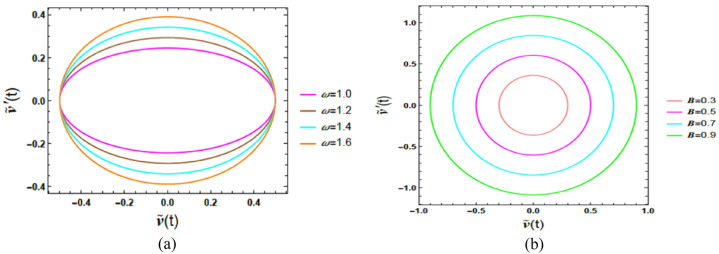
(**a**) Reflects the conduct of $\omega$ on the $\left( {\tilde{v},\,\dot{\tilde{v}}} \right)$ plane. (**b**) Reveals the impact of $B$ on the $\left( {\tilde{v},\,\dot{\tilde{v}}} \right)$ plane.

Phase plane analysis provides an insightful qualitative procedure in investigating the performance of second-order dynamical systems by representing their movement in a two-dimensional plane $\left( {\tilde{v},\,\dot{\tilde{v}}} \right)$, typically with one axis for displacement $\tilde{v}\left( t \right)$ and the other for its derivative $\dot{\tilde{v}}\left( t \right)$, velocity. Physically, closed trajectories correspond to periodic or sustained motion, spiralling paths indicate damped transients, and outward-diverging trajectories signify instability or energy growth. This representation clearly reveals the impact of system factors and ICs on long-term dynamics and is particularly useful for identifying nonlinear features such as limit cycles and stability transitions. Within this context, Fig. 9a and b depict the phase trajectories of the Rayleigh oscillator for diverse parameter amounts. All dimensionless parameters are kept identical to those employed in previous simulations, except for $B = 0.5,$ and $\beta_{R} = 0.52$.

In Fig. 9a, the phase plane $\left( {\tilde{v},\,\dot{\tilde{v}}} \right)$ shows how the frequency of the external forcing $\omega$ shapes the system’s movement. As $\omega$ escalated from 1 to 1.6, closed trajectories evolve from nearly circular to more oval and elongated shapes, with paths stretching horizontally and forming smoother, continuous loops. From a physical perspective, elevating $\omega$ modifies the rate at which energy is periodically injected into the oscillator. This geometric transformation reflects an increase in oscillation speed and a faster system response, indicating that the system completes its oscillatory cycles in shorter time intervals. The horizontal stretching of the trajectories signifies enhanced velocity variations relative to displacement, highlighting how higher excitation frequencies promote a more dynamic energy exchange within each oscillation cycle.

Figure 9b emphasizes the influence of varying the amplitude $B$ on the system’s conduct in the phase plane $\left( {\tilde{v},\,\dot{\tilde{v}}} \right)$. Physiologically, enlarging $B$ corresponds to supplying more energy to the system during each excitation cycle. As the amplitude increases from 3 to 9, the closed phase trajectories expand noticeably, indicating simultaneous growth in both displacement and speed magnitudes. This enlargement of the phase loops reflects an increase in the total mechanical energy of the system. Larger amplitudes cause broader trajectories, signifying stronger oscillatory motion and a more pronounced nonlinear response. Such performance is characteristic of Rayleigh-type oscillators, where amplitude-dependent mechanisms play a vital role in shaping the system’s energetic balance and long-term dynamics.

### Stable and unstable regimes of Case 2

**Fig. 10:**
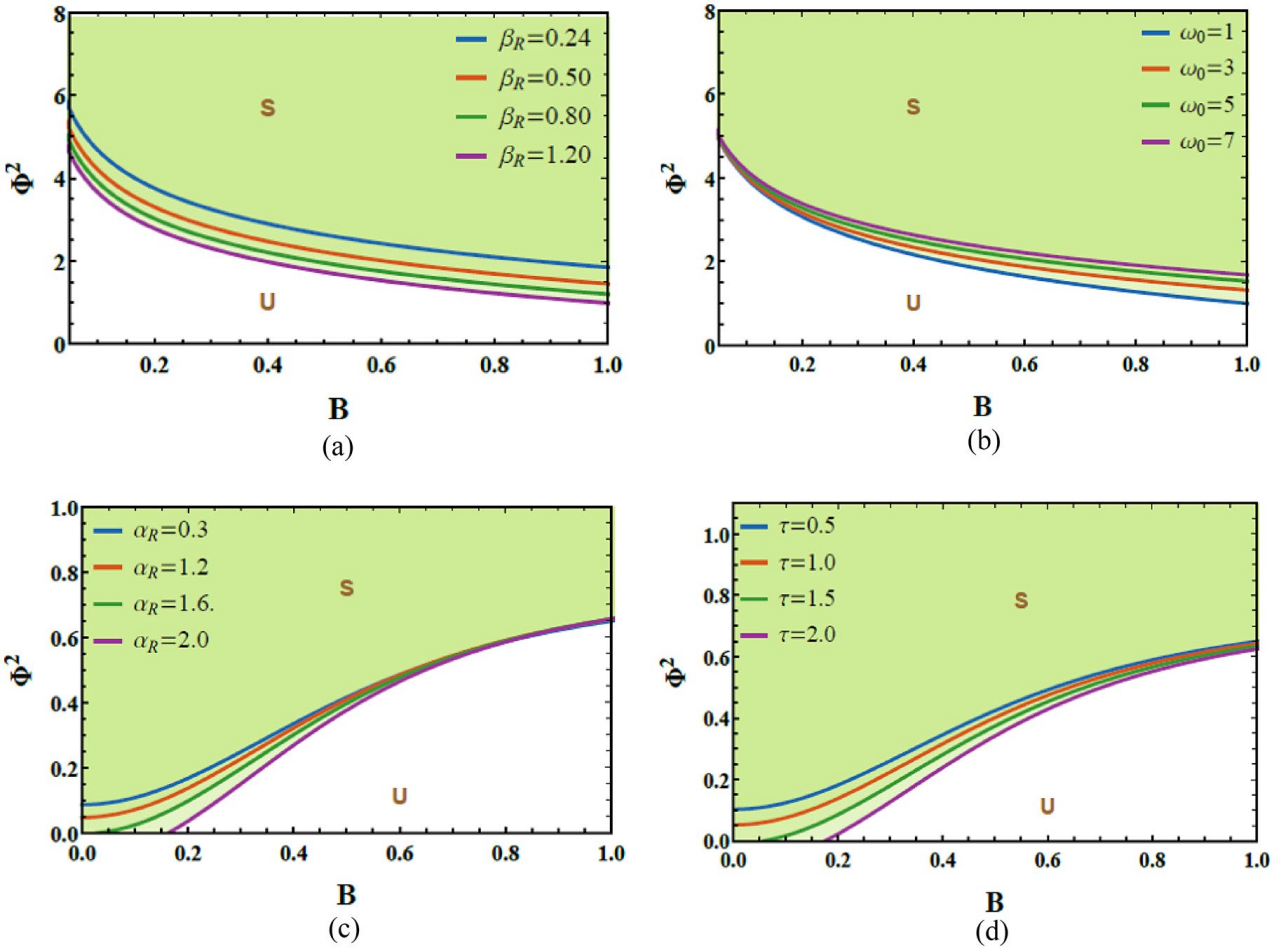
(**a**) Exposes the impact of $\beta_{R}$ on the stability diagram. (**b**) Shows the conduct of $\omega_{0}$ on the stability graph. (**c**) Reflects the impact of $\alpha_{R}$ on the stability scheme. (**d**) Exhibits the conduct of $\tau$ on the stability profile.

Figure 10a–d examine the stability characteristics associated with the restriction given in Eq. (33). These figures depict the variation of $\Phi^{2}$​ vs. $B$. The constraint $\Phi^{2} > 0$ assesses how the system responds under varying physical factors. The dimensionless factors are chosen as follows:

$$f = 0.2,$$$$\sigma = 0.5,\,$$$$\omega_{0} = 3.68,\omega = 0.02,\,\gamma = 0.005,\,\tau = 0.001,\,\,\alpha_{R} = 0.01,$$ $$g_{1} = 0.0002,\,\mu = 0.0001,$$ and $$\beta_{v} = 0.24.$$

Figure 10a demonstrates the stabilizing performance of the cubic damping factor $\beta_{R}$ on the stability scheme that plots $\Phi^{2}$ vs.$B$, As $\beta_{R}$ enhances from 0.24 to 1.20, the unstable regions progressively shrink, indicating a clear expansion of the system’s stable operating domain. From a physical viewpoint, stronger cubic damping enhances energy dissipation at larger oscillation amplitudes, suppressing excessive movement and limiting the growth of oscillatory energy. This nonlinear dissipation mechanism counteracts the destabilizing influences of excitation and delay, thereby promoting bounded oscillations and improving overall dynamic stability.

The influence of the natural frequency $\omega_{0}$ on stability fluctuations, represented by $\Phi^{2}$ vs.$B$, is depicted in Fig. 10b. It is found that as the natural frequency enlarges from 1.0 to 7.0, the stable areas progressively shrink, indicating a reduction in the overall stability domain. From a physical interpretation, a higher natural frequency causes faster oscillatory movement, which intensifies the interaction with parametric excitation and time-delayed feedback. Under such conditions, the system becomes more prone to resonance-like performance, where energy is supplied to the oscillator more efficiently than it can be dissipated by damping. Consequently, oscillatory energy accumulates, promoting amplitude growth and destabilizing the response. This explains the noted contraction of stable regions at elevated natural frequencies and underscores the importance of frequency tuning in maintaining the balance between energy input and dissipation in delayed nonlinear oscillatory systems.

Figure 10c demonstrates the stabilizing performance of the linear damping factor $\alpha_{R}$ on stability scheme, as $\alpha_{R}$ enhances, the unstable zones within the stability map progressively shrink, indicating an expansion of the stable operating domain. From a physical viewpoint, linear damping enhances energy dissipation by continuously extracting kinetic energy from the system’s movement. This suppresses oscillation growth and counteracts the energy injected through parametric excitation and delayed feedback. Consequently, higher damping levels limit amplitude amplification, promote bounded responses, and improve the overall robustness of the system against instability.

The impact of the time delay factor $\tau$ on the stability allocation is depicted in Fig. 10d. It is found that as the time delay factor escalates, the stable areas within the stability map gradually enlarge. From a physical perspective, an appropriate escalation in time delay modifies the phase relationship between the system response and the feedback forces, allowing delayed feedback to act in a compensatory manner rather than a destabilizing one. This phase adjustment can weaken resonance interactions and redistribute energy over time, thereby diminishing effective energy injection into the oscillator. As a consequence, oscillation amplitudes are restrained, and the system exhibits enhanced resistance to instability, causing an enlargement of the stable domain.

### PolarPlots analysis of case 2

**Fig. 11:**
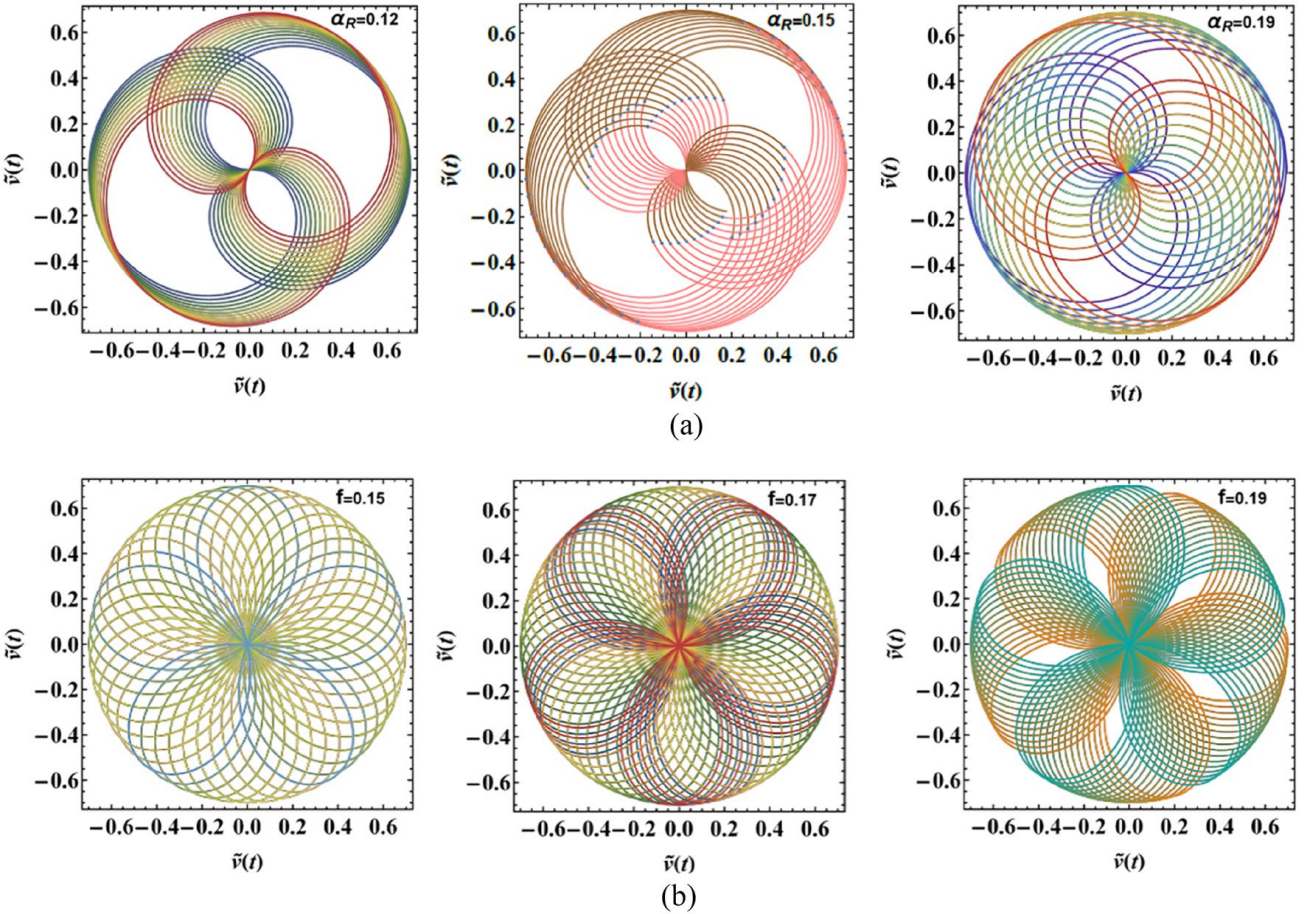
(**a**) Explains the PolarPlot schematic of $\hat{v}(t)$ vs.$\hat{v}(t)$ influenced by $\alpha_{R}$. (**b**) Depicts the PolarPlot scheme of $\hat{v}(t)$ vs.$\hat{v}(t)$ affected by $f$.

The PolarPlot diagrams presented in Fig. 11a and b expound the periodic rotational behavior of the system under diverse factor magnitudes associated with the solution $\hat{v}(t)$ given in Eq. (29). These plots provide a compact and physically meaningful representation of the oscillator’s response by mapping the displacement amplitude as a function of the angular phase. In the context of physical performance, the PolarPlots reveal how variations in the damping factor $\alpha_{R}$, as well as the external forcing amplitude $f$, govern the balance between energy injection and dissipation in the Rayleigh oscillator. Relying on the magnitude of these parameters, the trajectories form either closed loops, indicating bounded periodic motion and stable oscillations, or outward-spiraling paths, which signify progressive energy accumulation and the onset of instability. The geometric structure of these trajectories therefore serves as a direct indicator of the system’s rotational stability and the effectiveness of nonlinear damping mechanisms in regulating oscillatory energy.

## Bifurcation and poincaré maps

**Fig. 12:**
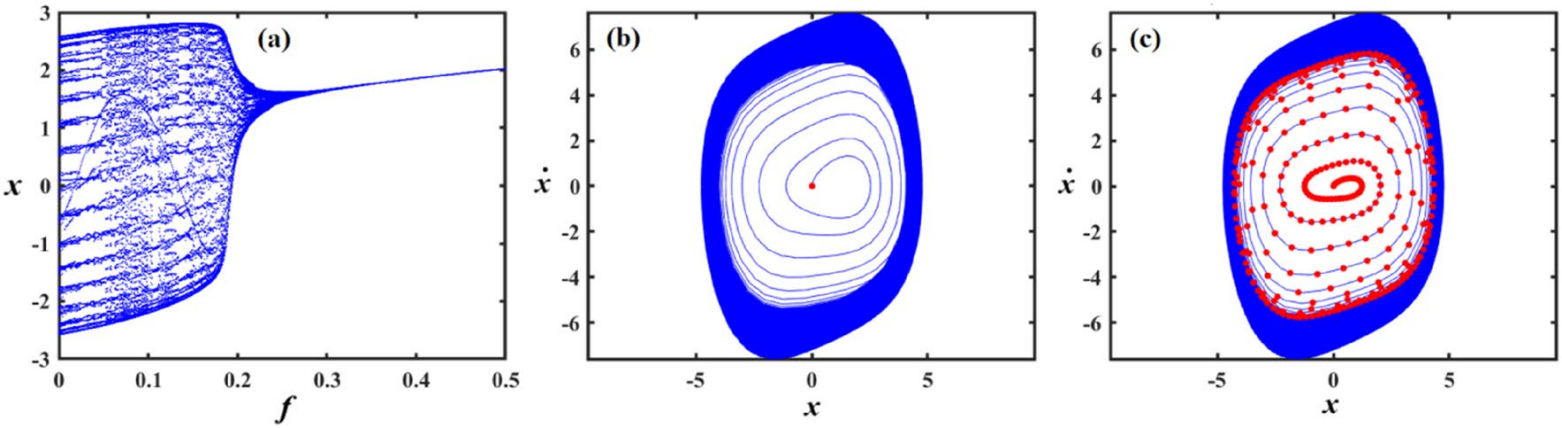
Exhibits (**a**) bifurcation diagram for $x$ versus $f$ and PM at (**b**)$f = 0.02$ and (**c**) at $f = 0.4$ when $\alpha_{v} = 1,\gamma = 0.1,\beta_{v} = 0.8,\mu = 0.05,\omega_{0} = 1,g_{1} = 0.9,\omega = 0.001,\Omega = 0.001$.

**Fig. 13:**
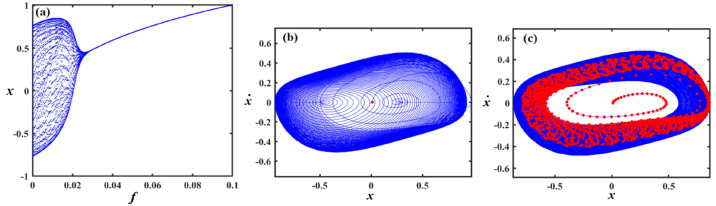
Expounds (**a**) bifurcation diagram for $x$ versus $f$ and PM at (**b**)$f = 0.06$ and (**c**) at $f = 0.01$ when $\alpha_{v} = 1,\gamma = 0.05,\beta_{v} = 0.8,\mu = 0.05,\omega_{0} = 1,g_{1} = 0.9,\omega = 0.001,\Omega = 0.001$.

**Fig. 14:**
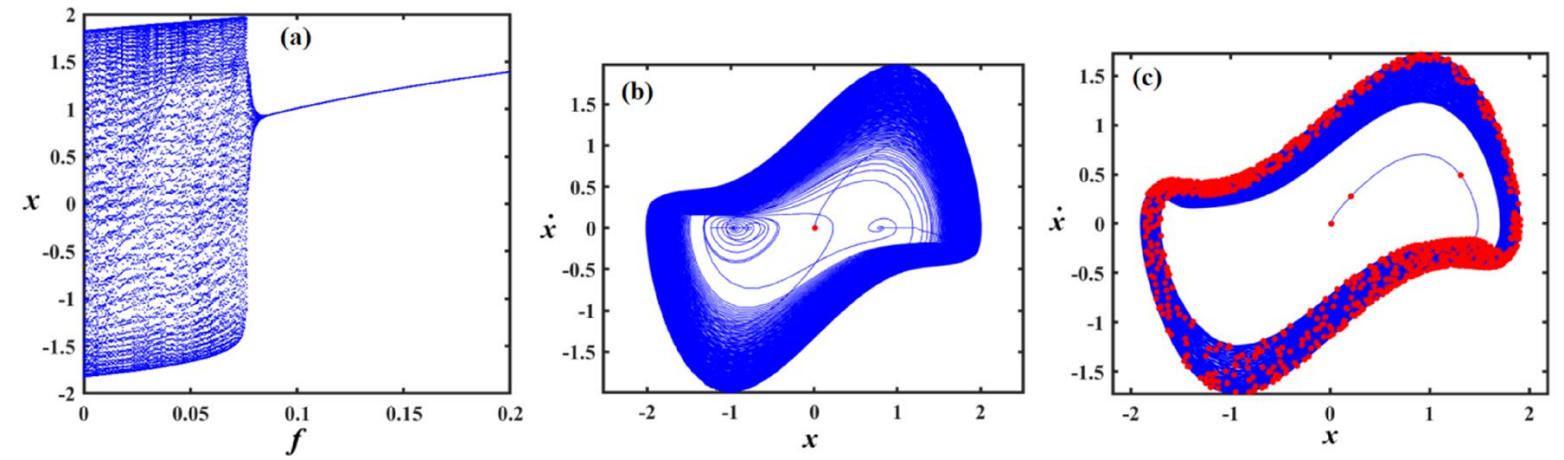
Describes (**a**) bifurcation diagram for $x$ versus $f$ and PM at (**b**)$f = 0.15$ and (**c**) at $f = 0.04$ when $\alpha_{v} = 1.5,\gamma = 0.05,\beta_{v} = 0.8,\mu = 0.05,\omega_{0} = 1,g_{1} = 0.9,\omega = 0.001,\Omega = 0.001$.

**Fig. 15:**
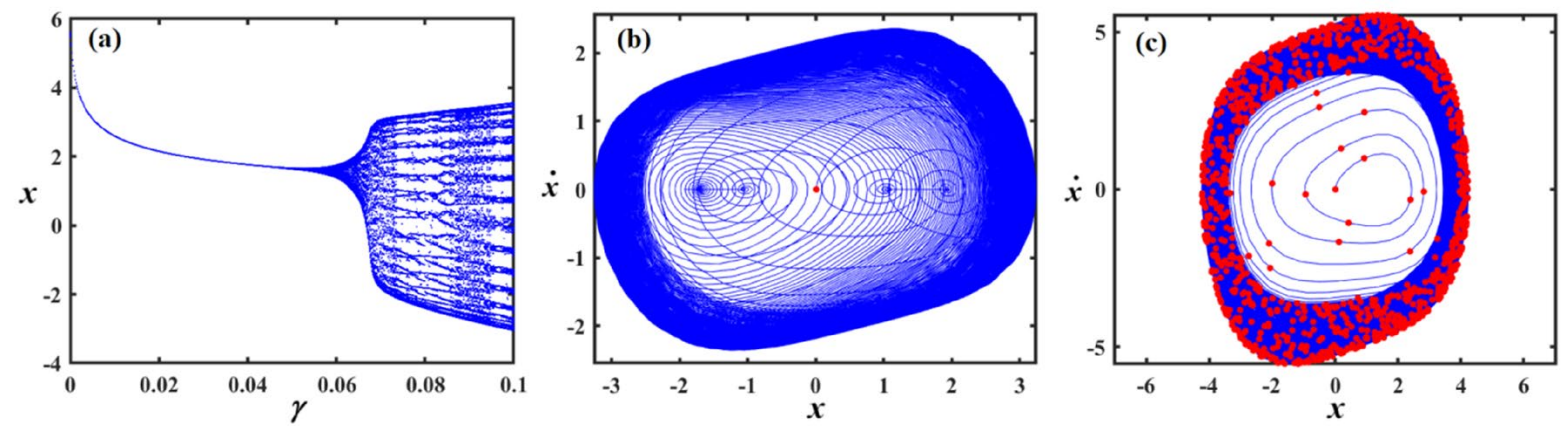
Outlines (**a**) bifurcation diagram for $x$ versus $\gamma$ and Poincaré map at (**b**) $\gamma = 0.03$ and (**c**) at $\gamma = 0.08$ when $\alpha_{v} = 1,f = 0.3,\beta_{v} = 0.08,\mu = 0.05,\omega_{0} = 1,g_{1} = 0.9,\omega = 0.001,\Omega = 0.001$.

**Fig. 16:**
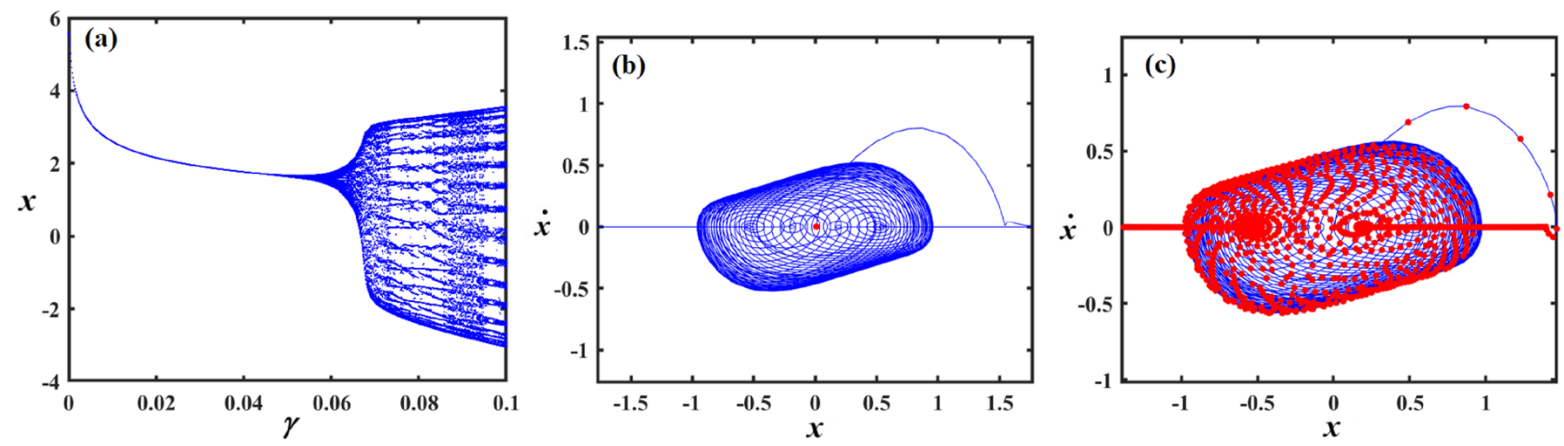
Reflects (**a**) bifurcation diagram for $x$ versus $\gamma$ and PM at (**b**) $\gamma = 0.04$ and (**c**) at $\gamma = 0.09$ when $\alpha_{v} = 1,f = 0.3,\beta_{v} = 0.8,\mu = 0.05,\omega_{0} = 1,g_{1} = 0.9,\omega = 0.001,\Omega = 0.001$.

**Fig. 17:**
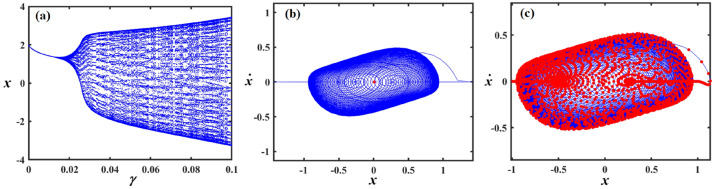
Shows (**a**) bifurcation diagram for $x$ versus $\gamma$ and PM at (**b**) $\gamma = 0.01$ and (**c**) at $\gamma = 0.05$ when $\alpha_{v} = 1,f = 0.1,\beta_{v} = 0.8,\mu = 0.05,\omega_{0} = 1,g_{1} = 0.9,\omega = 0.001,\Omega = 0.001$.

**Fig. 18:**
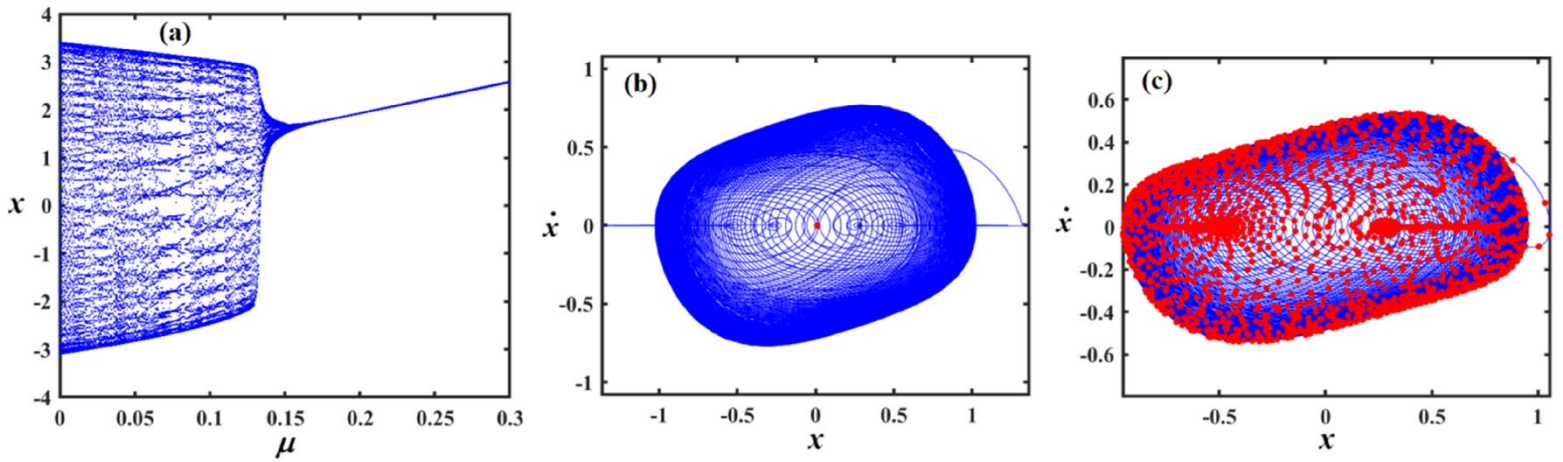
Exposes (**a**) bifurcation diagram for $x$ versus $\mu$ and PM at (**b**) $\mu = 0.18$ and (**c**) at $\mu = 0.04$ when $\alpha_{v} = 1,f = 0.1,\beta_{v} = 0.8,\gamma = 0.1,\omega_{0} = 1,g_{1} = 0.9,\omega = 0.001,\Omega = 0.001$.

**Fig. 19:**
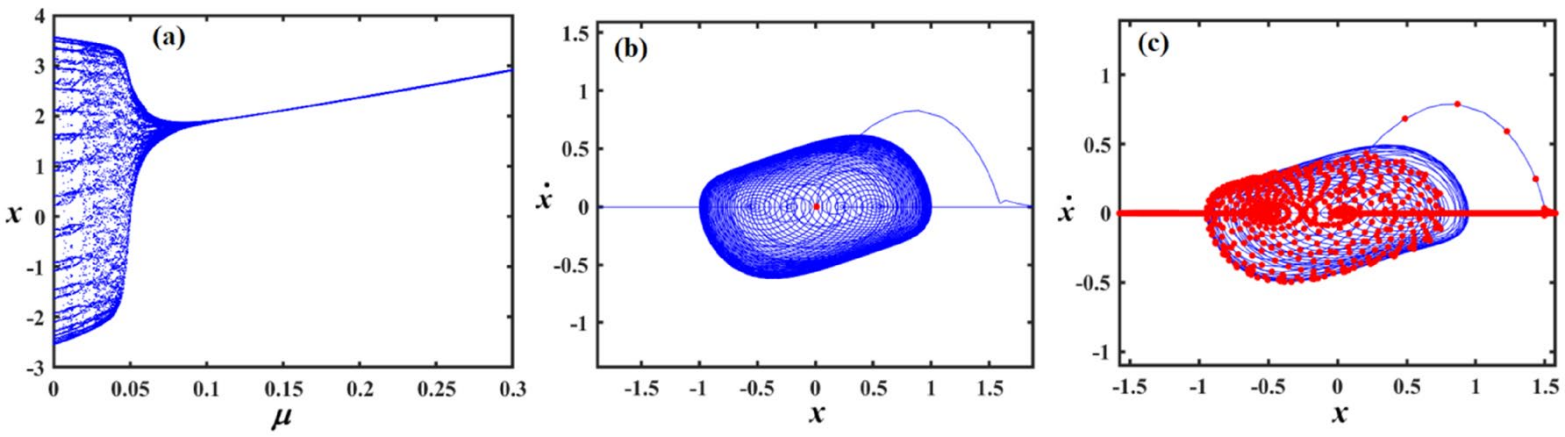
Demonstrates (**a**) bifurcation diagram for $x$ versus $\mu$ and PM at (**b**) $\mu = 0.2$ and (**c**) at $\mu = 0.03$ when $\alpha_{v} = 1,f = 0.3,\beta_{v} = 0.8,\gamma = 0.05,\omega_{0} = 1,g_{1} = 0.9,\omega = 0.001,\Omega = 0.001$.

This section focuses on the analysis of the graphical representations of bifurcation curves, which elucidate the structural metamorphosis of dynamical systems under parametric variations. These visualizations capture transformative moments where systemic performance qualitatively diverges, revealing emergent equilibrium configurations and periodic trajectories. PMs complement these analyses by quantifying systemic stability and chaotic tendencies. Collectively, these maps provide sophisticated insights into complex dynamical transitions^46–48^. Therefore, we can recall Eq. (4) for the case of a damped van der Pol oscillator, in which the bifurcation diagram of the variable $x$ versus $f$ was simulated in Figs. 12, 13, 14, 15, 16, 17, 18 and 19.

Figure 12a shows a bifurcation diagram where the system transitions from chaotic performance to a regular regime as $f$ increases. In the first interval, at $f \in [0,0.27]$ , the system’s performance is chaotic, demonstrating highly sensitive and irregular dynamics, indicating a transition toward a periodic pattern. Within the second interval, at $f \in (0.27,0.5]$ the system exhibits a periodic pattern. Figure 12b, c reflect the phase portraits and PM for diverse values of $f$. In these plots, the blue curves demonstrate the behavioural trajectory within the phase domain, representing the phase portraits, while the red dots denote the PM, offering information about the system’s stability and periodic performance. In part (b), when $f = 0.02$ a single red dot located near the centre suggests a fixed point in the PM, it indicates the system is likely in a stable periodic or quasi-periodic state with a single intersection per cycle. In part (c) at $f = 0.4$, multiple red dots form a spiral pattern converging inward. This suggests the system is a chaotic attractor.

Figure 13a shows a bifurcation diagram where the system transitions from chaotic behavior to a regular regime as the forcing amplitude $f$ escalates. In the first interval, at the forcing amplitude $f \in \left[ {0,\,0.03} \right]$, the system’s conduct is chaotic, demonstrating highly sensitive and irregular dynamics, indicating a transition toward a periodic pattern. Within the second interval, at $f \in \left[ {0.03,\,0.1} \right]$, the system’s performance becomes a periodic pattern. Subplots (b) and (c) reflect the phase portraits and PM for diverse measures of the forcing amplitude. In (b), at $f = 0.06$ the system exhibits a stable periodic orbit with a dense, nested trajectory structure and red markers indicating fixed points or attractors. In (c), at $f = 0.01$ the phase space becomes more complex, with scattered red points suggesting chaotic dynamics and the breakdown of the periodic orbit observed in (b).

Figure 14 focuses on the analysis of a nonlinear system, showing its dynamic behavior under increased forcing. Subplot (a) presents a bifurcation diagram of the system’s displacement $x$ versus the forcing amplitude $f$, revealing a more extended chaotic region compared to Fig. 13, with a denser pattern of bifurcations before the system stabilizes into a periodic state. Subplot (b) shows a phase portrait with a spiral trajectory converging toward a periodic orbit, and a red point indicating an attractor, suggesting a weakly stable limit cycle. Subplot (c) displays a more complex attractor, with red points highlighting a chaotic set and blue lines showing the trajectory approaching this strange attractor.

Figure 15a expounds a bifurcation diagram showing how the variable $x$ evolves with respect to the parameter $\gamma$, revealing period-doubling bifurcations at $f \in [0,0.06]$ and the onset of chaos $\gamma$ at $f \in (0.06,0.1]$. Panel (b) shows a phase portrait in the $(x,\dot{x})$ plane, illustrating the system’s attractor structure with closed curves around equilibrium points, suggesting quasi-periodic or multi-stable behavior. Panel (c) also presents a phase portrait, likely under a diverse parameter set or with perturbations, highlighting a strange attractor with a dense, chaotic trajectory in blue and overlaid red points representing a Poincaré section, which maps intersections of trajectories with a lower-dimensional subspace, indicating chaotic dynamics.

Figure 16a shows the bifurcation diagram of $x$ via the parameter $\gamma$, featuring a classic period-doubling route to chaos as $\gamma$ increases beyond a critical threshold. Panel (b) presents a phase portrait, where the trajectories spiral inward toward a central limit cycle or fixed point, indicating stable, periodic behavior. In panel (c), the same phase space is shown, but with red dots marking a Poincaré section that captures the intersections of the trajectory with a defined surface, highlighting the underlying structure of the attractor. The escalated density and apparent irregularity of red points suggest the presence of a chaotic attractor.

Part (*a*) in Fig. 17 shows a bifurcation diagram of the variable $x$ vs. the parameter $\gamma$, with the system rapidly transitioning into a chaotic state after a small increase in $\gamma$, characterized by an intricate structure of period-doubling bifurcations and dense branches. Part (b) shows the phase portrait featuring a closed-loop attractor with densely packed trajectories spiralling around a central point, indicating a stable or quasi-periodic regime. Part (c) overlays the same phase space with a Poincaré section shown in red, capturing intersections of the trajectory with a chosen surface, revealing a more complex internal structure with signs of deterministic chaos, as evidenced by the irregular and scattered distribution of points. The increasing density and pattern complexity compared to previous figures suggest the system is operating deeper within a chaotic regime.

Figure 18a shows a bifurcation diagram of the variable $x$ vs. the parameter $\mu$, where the system transitions from chaotic behavior to a regular regime as $\mu$ escalates. In the first interval, for ($\mu \in [0,0.15]$), the system exhibits chaotic performance characterized by high sensitivity and irregularity, suggesting a shift toward periodic dynamics. During the second interval, as $\mu \in (0.15,0.3]$, the system evolves into a distinct periodic pattern. Part (b), for ($\mu = 0.18$) exhibits the phase portrait, highlighting a closed-loop attractor where trajectories tightly spiral around a central point, suggesting a stable or quasi-periodic system. In part (c), (at $\mu = 0.04$) the same phase space is depicted with an overlaid Poincaré section in red, marking where the trajectory intersects a selected surface. This reveals a more intricate internal pattern, with the scattered and irregular points indicating the presence of deterministic chaos.

In Fig. 19a, the system shifts from chaotic dynamics to more regular behavior. Initially, at $\mu \in [0,0.1]$, the system displays chaotic characteristics, marked by high sensitivity and unpredictability, indicating a move toward periodic performance. Later, at $\mu \in (0.1,0.3]$, it clearly settles into a periodic regime portion (b) at $\mu = 0.2$ displays a stable or quasi-periodic phase portrait characterized by spiraling trajectories, whereas portion (c) at $\mu = 0.03$ indicates deterministic chaos through a Poincaré section including dispersed intersection points.

**Fig. 20:**
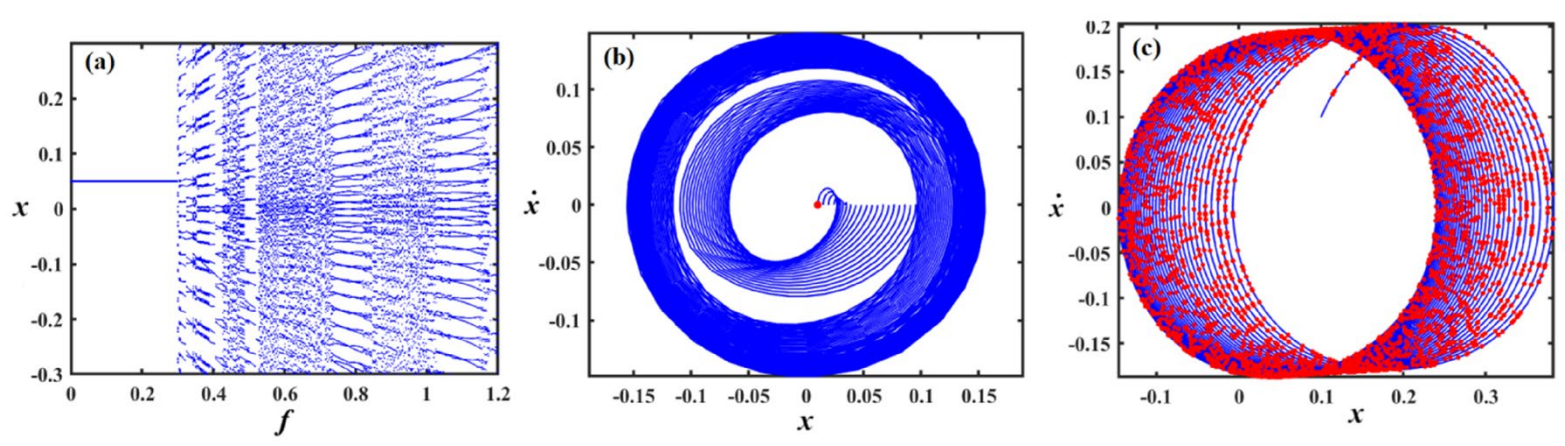
Depicts (**a**) bifurcation diagram for $x$ versus $f$ and PM at (**b**) $f = 0.03$ and (**c**) at $f = 1$ when $\alpha_{R} = 0.01,\gamma = 0.04,\beta_{R} = 1.2,\mu = 0.08,\omega_{0} = 1,g_{1} = 0.25,\omega = 1.2,\Omega = 1.2$

Regarding the bifurcation diagrams for the considered Eq. (19), this relates to the damped Rayleigh oscillator. Figure 20 exposes the nonlinear dynamic behavior of a forced oscillator system as the forcing amplitude $f$ varies. Subplot (a) presents a bifurcation diagram showing how the system’s steady-state displacement $x$ evolves with escalating $f$, transitioning from a stable periodic solution (single line) at low forcing $f \in [0,0.3]$ to complex, chaotic dynamics (dense scattered points) beyond $f \in (0.3,1.2]$. Subplots (b) and (c) display phase portraits in the $(x,\dot{x})$ plane: (b) shows a stable periodic orbit with a clear spiral trajectory converging to a limit cycle, while (c) reveals chaotic motion with a scattered and irregular Poincaré section (red dots), corresponding to the chaotic regime in (*a*). Together, the plots capture the system’s route to chaos through bifurcation and provide insight into its periodic and chaotic attractors.

**Fig. 21:**
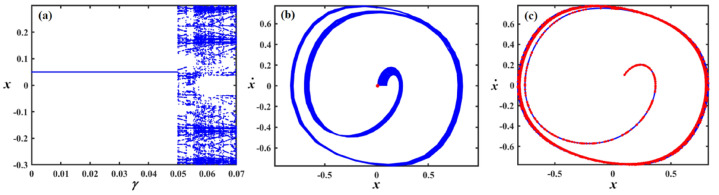
Demonstrates (**a**) bifurcation diagram for $x$ versus $\gamma$ and PM at (**b**) $\gamma = 0.01$ and (**c**) at $\gamma = 0.06$ when $\alpha_{R} = 0.5,f = 0.2,\beta_{R} = 1.2,\mu = 0.2,\omega_{0} = 1,g_{1} = 0.2,\omega = 1,\Omega = 1$.

Figure 21 reflects the nonlinear dynamics of a system under variation of the parameter $\gamma$. In subplot (a), the bifurcation diagram shows how the displacement $x$ evolves with increasing $\gamma$, transitioning from a stable fixed point (a single horizontal line for $\gamma \in [0,0.05]$) to chaotic behavior (scattered points) for $\gamma \in (0.05,0.07]$, indicating a route to chaos as $\gamma$ increases. Subplot (b) depicts a phase portrait for the periodic regime, where the trajectory spirals inward toward a stable fixed point or periodic attractor, with red dots likely representing Poincaré section points. Subplot (c) shows a phase portrait corresponding to the chaotic regime, where the trajectory fills a broader region of phase space and the red Poincaré points are scattered, indicating a loss of periodicity and the onset of chaos. Collectively, these plots illustrate the transition from stable to chaotic dynamics as a function of $\gamma$.

**Fig. 22:**
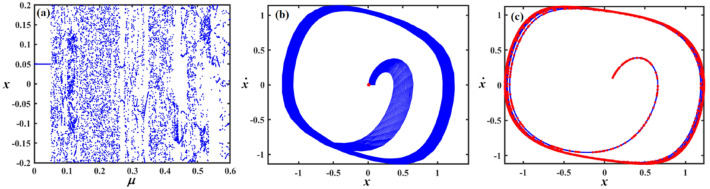
Highlights (**a**) bifurcation diagram for $x$ versus $\mu$ and PM at (**b**) $\mu = 0.002$ and (**c**) at $\mu = 0.3$ when $\alpha_{R} = 1.2,f = 0.2,\beta_{R} = 1.2,\gamma = 0.05,\omega_{0} = 1,g_{1} = 0.2,\omega = 1,\Omega = 1$.

Figure 22 shows a bifurcation diagram of the variable $x$ vs. the parameter $\mu$, where the system transitions from regular behavior to a chaotic regime as $\mu$ increases. During the initial interval, at $\mu \in [0,0.05]$, the system demonstrates a stable fixed point, depicted by a singular horizontal line. In the second interval, at $\mu \in (0.05,0.6]$, the system transitions into chaotic behavior, characterized by high sensitivity and irregularity. Subplot (b) presents a phase portrait with a spiral-like trajectory converging to a distorted limit cycle, showing a periodic attractor under a specific $\mu$ value, with red dots marking the Poincaré section. In subplot (c), the phase portrait reveals a chaotic attractor with scattered red Poincaré points filling a wider region of phase space, highlighting the loss of regularity. Overall, this figure illustrates that as $\mu$ varies, the system remains largely in a chaotic regime, with complex attractor structures and sensitive dependence on ICs.

It must be noted that the transient phases in Figs. 12, 13, 14, 15, 16, 17, 18, 19, 20, 21 and 22 of a chaotic plot are not merely preliminary stages to be ignored in a vibrating dynamical system. It has a wealth of dynamic information. Compared to the steady state, it provides a more complete picture of the system’s nonlinear nature. It creates a phase space map of the safe and dangerous operation areas.

Ignoring transients in practical engineering might result in surprises and failures since it ignores the system’s path and concentrates exclusively on its destination. For nonlinear vibrating systems to be designed robustly, monitored for health, and operated safely, an understanding of chaotic transients is essential.

It is noted from Fig. 13a that, at $f < 0.22$, a dense cloud-like structure expresses a chaotic region. On the other side, at $f > 0.22$, a single smooth branch is observed that reveals a stable periodic motion. Figure 13b and c have blue clouds and red dots. They illustrate a strange attractor and a periodic orbit approached for higher forcing, respectively.

**Fig. 23:**
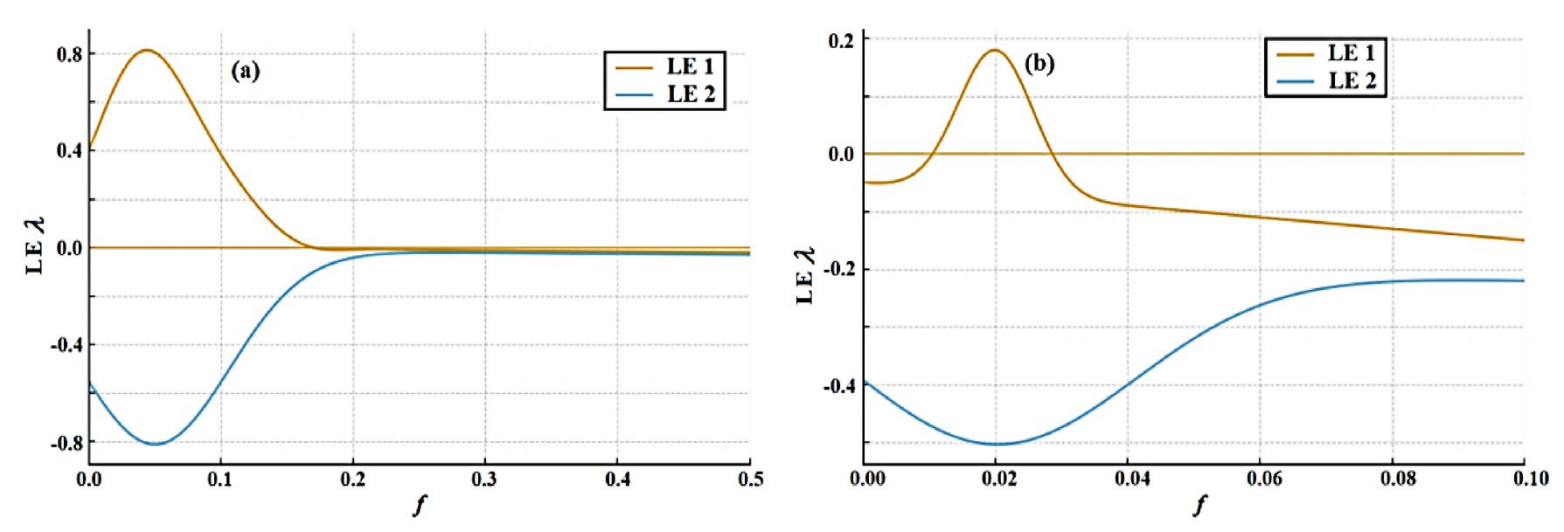
Presents LE via the parameter $f$ according to Figs. 12 and 13 for case 1.

Based on the plotted curves in Fig. 23a, one can observe that the performance of LE $\lambda$ is described as follows: at $0 < f < 0.20$, trajectories diverge, and strong irregular mixing in the Poincaré map is noted. When $\lambda > 0$, a chaotic region is observed.

The transition area is found at $\left( { \approx \,0.20 - 0.23} \right)$, which expresses intermittency and unstable periodic windows, while $\lambda$ fluctuates around zero. As $\lambda$ approaches $0$ (in the right direction), light chaos is noted. After that, $\lambda$ crosses to negative, this corresponds exactly to the merging of branches in Fig. 13a. Clean, single attractor and no exponential divergence are observed for post-transition (i.e.,$f > 0.23$). At $\lambda < 0$, stable periodic motion is obtained. As forcing grows, the periodic orbit amplitude expands but is still stable.

As stated before, one can analyze the LE curves in Fig. 23b as follows: When the forcing amplitude is $f \in [0,0.015]$, the dynamical performance of the examined system is periodic, and the LE curve lies in the negative region. Weak chaos is found at $f \in [0.018,0.025]$ where LE has a slightly positive area, while the dynamical conduct may be periodic or quasi-periodic when $f > 0.03$ in which the LE curve lies in a negative domain.

**Fig. 24:**
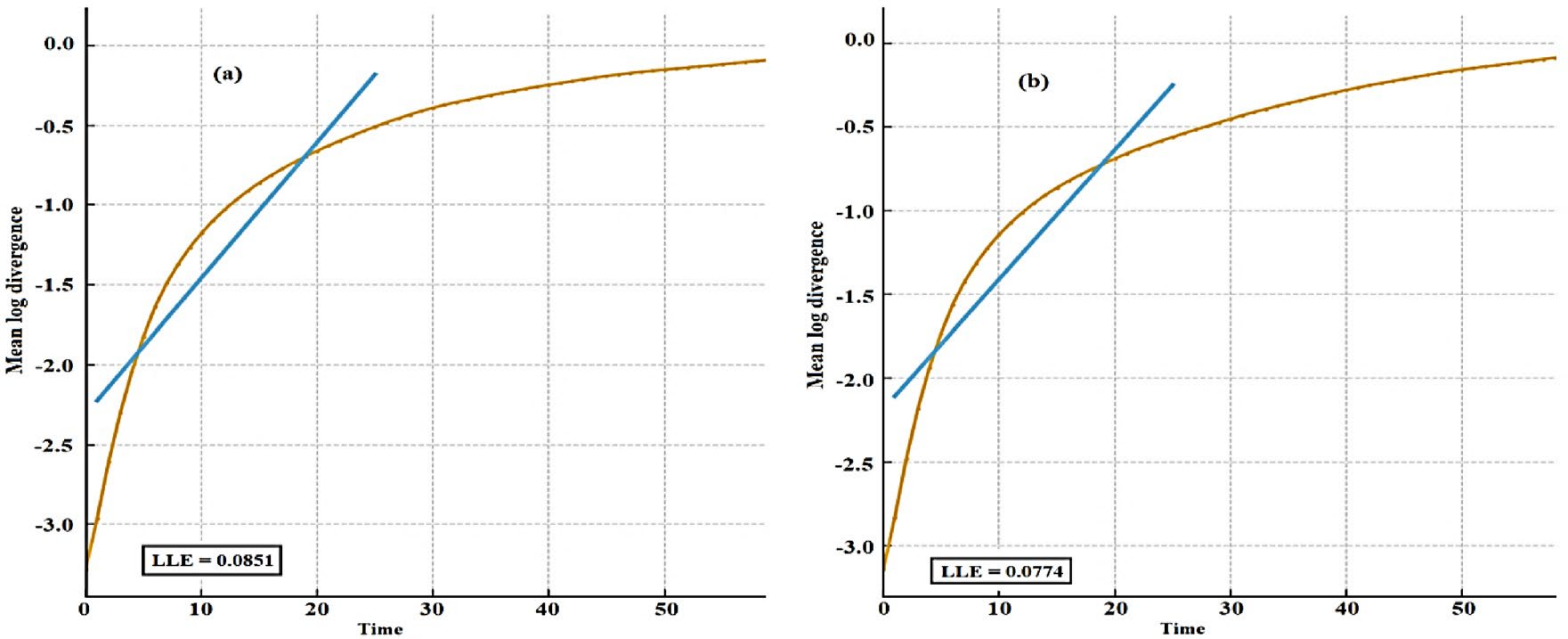
Demonstrates mean log divergence via time for the parameters $\gamma$ and $\mu$ for case 1.

Two LE curves, each of which represents the mean logarithmic divergence of adjacent trajectories taken from Figs. 16 and 19 are included in the combined Fig. 24. The explanation for each is given below.

The mean log divergence curve climbs roughly linearly throughout the first segment, as seen in Fig. 16. The largest Lyapunov exponent (LLE), or fitted slope, is positive; the precise numerical value can be seen on your figure. The divergence saturates after the linear growth zone, which is characteristic of bounded attractors.

The following is the physical interpretation of the curves in Fig. 24a Chaotic dynamics and sensitive dependence on initial circumstances are indicated by a positive LE. This indicates that the motion depicted in Fig. 16 is chaotic, which is in line with the original figure’s bifurcation structure, complicated attractor form, and wide, fuzzy Poincaré section. As a consequence, it verifies that, for the selected parameter, the system in Fig. 16 is functioning in a chaotic regime.

Figure 19 shows that the fitted slope of the LLE is extremely close to zero but still slightly positive, and the mean log divergence curve rises much more slowly. Compared to Fig. 16, the fitted line has a substantially smaller vertical scale. Consequently, the following physical illustration of this behavior is possible: As seen in the original Fig. 19, the modest near-zero LE indicates quasi-periodic or weakly chaotic activity, no strong exponential divergence, and the attractor is almost a smooth closed torus, see Fig. 24b. Therefore, depending on the precise value, the motion is either quasi-periodic or the beginning of mild chaos rather than severely chaotic. A modest positive LLE is frequently associated with intermittent dynamics, extremely weakly chaotic attractors, and the start of torus collapse. The extracted exponent and the original figure’s visual appearance suggest that quasi-periodicity is the most likely dominating behavior.

According to calculations, the LLE of Fig. 16 is nearly (positive and obviously over zero), and the exponential divergence of neighboring trajectories is an indication of deterministic chaos, see Fig. 24a. In contrast, Fig. 19 's value is near zero but falls between $0.07$ and $0.08$ (reduced effective slope in the fitted range). It shows near-torus behavior that is not strongly chaotic, weak chaos, and quasi-periodicity, see Fig. 24b.

Based on the calculated Figs. 20, 21 and 22, the LLE for the three parameter sweeps ($f,\gamma ,$ and $\mu$) has been clearly and systematically analyzed. We shall explain how the sign and structure of $\lambda$-periodic, quasi-periodic, or chaotic regimes, as well as transitions between them, imply dynamical performance.

**Fig. 25:**
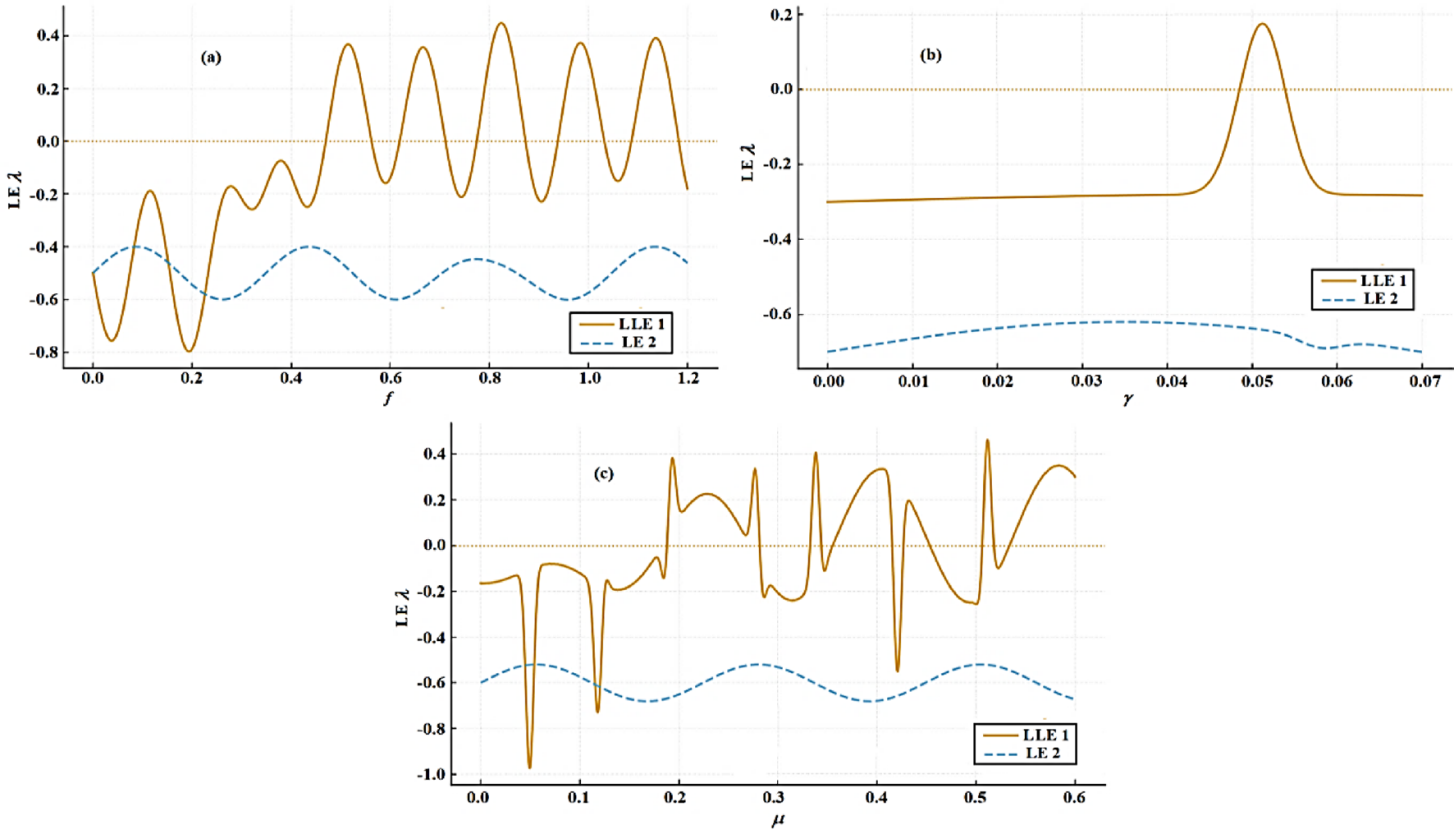
Shows LE via the parameters $f,\gamma ,$ and $\mu$ for case 2.

Examining $\lambda$ vs.$f$, as in Fig. 24a, can be stated as follows: The primary findings when $f \in [0,1.2]$ are: The greatest LLE $\lambda < 0$ for tiny $f$ results in stable periodic orbits. $\lambda$ exhibits oscillatory activity with bands of positive values when f rises above around $0.35$. On the other hand, $\lambda > 0$ indicates chaos in the system. As seen in Fig. 25a, these chaotic zones occur in windows that are separated by areas where $\lambda$ declines once more ($\lambda < 0$).

One can examine the value of the $\gamma$ parameter as $\gamma \in [0,0.07]$ in order to analyze $\lambda$ vs.$\gamma$, as shown in Fig. 25b. The primary findings are: For nearly the whole interval, $\lambda$ is negative, resulting in mostly non-chaotic dynamics. A very small peak when $\lambda$ turns positive at $\gamma \approx 0.05$. A little chaotic window embedded in a largely regular regime is suggested by this peak’s sharpness and localization. The system under study exhibits characteristics of intermittency-induced chaos, a tiny parameter window where the stable limit cycle becomes unstable, and potentially a torus breakdown or saddle-node bifurcation of periodic orbits. The system has low sensitivity to initial conditions ($\lambda < 0$) for most $\gamma$ amounts and strong sensitivity only close to $\gamma \approx 0.05$ because the chaos only occurs in a limited interval.

Plotting $\lambda$ vs. $\mu$ as shown in Fig. 25c allows one to analyze the performance of the system based on the parameter $\mu$. Over the whole $\mu$ range, $\lambda$ repeatedly oscillates between negative and positive values when $\mu \ge [0,0.6]$. There are numerous narrow chaotic windows ($\lambda > 0$ spikes) where the total chaos is higher than that of the γ-sweep. The system as a whole exhibits the characteristics of a multi-stable nonlinear oscillator that alternates between chaotic and regular performance as $\mu$ changes.

## Conclusions insights

This work examined a nonlinear Mathieu oscillator with time-delay feedback using a unified analytical framework that integrated NPA and HFF, in contrast with any traditional perturbation techniques. It successfully analyses the impacts of self-excitation, parametric excitation, external forcing, and time-delay feedback on dynamics, deriving analytical expressions of amplitude-frequency relationships and stability criteria, which were validated through NSs. The outcomes demonstrate that time delay and nonlinear excitation mechanisms play a conclusive role in reshaping the stability landscape of the system, giving rise to rich dynamical performances including stable periodic oscillations, parametric instabilities, and chaotic responses. In particular, the delay parameter was shown to significantly modify instability tongues and stability thresholds, highlighting its critical influence on nonlinear vibration control. The principal findings of this work can be summarized as follows:

1. The stability characteristics of the excited nonlinear Mathieu system were systematically analyzed for both van der Pol and Rayleigh oscillator configurations, revealing distinct stability mechanisms associated with each model.
2. For the van der Pol oscillator, escalating the natural frequency enhances system stability, whereas the nonlinear damping factor exhibits a destabilizing performance by promoting self-excited oscillations.
3. In contrast, for the Rayleigh oscillator, the cubic nonlinear damping term plays a stabilizing role by suppressing large-amplitude oscillations, while elevating the natural frequency diminishes the stable operating domain and promotes instability.
4. A systematic comparison between analytical predictions (HFF-NPA) and numerical simulations was conducted employing tabulated data and graphical diagnostics, confirming the accuracy and robustness of the proposed approach.
5. Additional nonlinear diagnostic tools, involving bifurcation schemes, PMs, and Les, were applied to further characterize transitions between periodic, quasi-periodic, and chaotic regimes.

Finally, this work uncovers previously unexplored stability transitions and delay-induced instability mechanisms in nonlinear Mathieu oscillators with time delay feedback. By relying on the NPA framework, the study provides verifiable stability boundaries and dynamic regimes that are not accessible through conventional approximation-based techniques.

### The importance and applications of this work

The analysis of excited nonlinear Mathieu oscillators with time delay is of considerable importance in a wide range of applications, including mechanical vibration absorbers, electrical and electromechanical systems, delayed feedback control, and biological rhythm modeling. Time delay naturally arises from signal transmission, sensing, and actuation processes, while Mathieu excitation captures periodic parametric influences commonly encountered in engineering systems. The current findings offer practical guidelines for tuning system parameters to suppress instability or exploit controlled oscillations, thereby contributing to the design and stabilization of nonlinear dynamical systems subject to delayed feedback.

### Future work

Future research will extend the proposed NPA–HFF framework to multi-degree-of-freedom and coupled oscillator systems, as well as to stochastic and fractional-order models. These extensions aim to establish the methodology as a versatile analytical and computational tool for complex nonlinear dynamical systems encountered in applied mathematics, control engineering, and advanced mechanical design.

## Data Availability

All data generated or analyzed during this study are included in this published article.
